# Proteomic identification and histopathological validation of candidate biomarkers CA1, HBB, Torsin A, and *α*-Tubulin in gingivo-buccal oral squamous cell carcinoma

**DOI:** 10.3389/fmed.2026.1843924

**Published:** 2026-08-31

**Authors:** Vidyarani Shyamsundar, Soundarya Ravindran, Arvind Krishnamurthy, Divyambika Catakapatri Venugopal, Yasasve Madhavan, Ananthi Sivagnanam, Anju Mayadevi Nair, Sanjana Vimal, Pallavi Kesavan, N. Senthil Subramaniyan, N. Aravindha Babu, Vijayalakshmi Ramshankar

**Affiliations:** 1 Department of Cancer Biology and Molecular Diagnostics, Cancer Institute (WIA), Chennai, India; 2 Department of Oral and Maxillofacial Pathology, Sree Balaji Dental College and Hospital, Chennai, India; 3 Department of Surgical Oncology,Cancer Institute (WIA), Chennai, India; 4 Department of Oral Medicine and Radiology, Sri Ramachandra Dental College and Hospital, Sri Ramachandra Institute of Higher Education and Research (DU), Chennai, India

**Keywords:** biomarkers, CA1, gingivo-buccal oral squamous cell carcinoma, HBB, immunohistochemistry, MALDI-TOF, OSCC, proteomics

## Abstract

**Background:**

Gingivo-buccal oral squamous cell carcinoma (GB-OSCC) represents one of the most prevalent tobacco-associated malignancies in India and is frequently diagnosed at advanced stages with poor clinical outcomes. Despite the high disease burden, validated specific biomarkers for gingivo-buccal cancers remain limited. The current study aimed to identify and validate protein biomarkers associated with disease progression and aggressive pathological features in GB-OSCC using an integrated proteomic and immunohistochemical (IHC) approach.

**Methods:**

Proteomic discovery analysis was performed on pooled fresh-frozen tumor tissues obtained from 40 histologically confirmed GB-OSCC patients categorized into four groups based on clinical stage and treatment outcome: early-stage no evidence of disease (NED), early-stage failure, advanced-stage NED, and advanced-stage failure (*n* = 10/group). Differential protein expression profiling was carried out using two-dimensional gel electrophoresis (2DE) followed by matrix-assisted laser desorption/ionization-time of flight mass spectrometry. Functional enrichment and protein interaction analyses were performed using PANTHER and STRING databases. Four candidate proteins—Carbonic Anhydrase 1 (CA1), Hemoglobin subunit beta (HBB), Torsin A, and *α*-Tubulin—were selected for validation by immunohistochemistry in retrospective formalin-fixed paraffin-embedded tissues comprising healthy controls, oral epithelial dysplasia, and GB-OSCC cases (*n* = 125).

**Results:**

Proteomic analysis identified 37 differentially upregulated proteins associated with tumor progression, cytoskeletal remodeling, metabolic regulation, and angiogenesis. STRING network analysis demonstrated enrichment of proteins involved in glycolysis, apoptosis signaling, keratinization, and cellular regulatory pathways. IHC validation revealed progressive overexpression of CA1, HBB, Torsin A, and *α*-Tubulin across the spectrum from healthy mucosa to dysplasia and invasive carcinoma. Increased expression of CA1 and HBB showed significant associations with tumor size, degree of differentiation, muscle invasion, lymphovascular invasion, depth of invasion, invasive tumor front characteristics, and tumor budding. Torsin A and *α*-Tubulin expression correlated significantly with advanced histopathological grade, invasive behavior, and aggressive tumor phenotypes. Receiver operating characteristic (ROC) curve analysis showed that HBB and CA1 have significant area under the curve (AUC) indicating good discriminatory ability for SCC detection.

**Conclusion:**

This study identifies and validates a subsite-specific protein biomarker panel for gingivo-buccal OSCC in an Indian cohort. The combined overexpression of CA1, HBB, Torsin A, and *α*-Tubulin demonstrates potential utility in identifying aggressive disease biology and may contribute toward improved prognostic stratification and translational biomarker development in tobacco-associated oral cancers. Larger multicentric studies are warranted to establish their clinical applicability.

## 1 Introduction

Oral squamous cell carcinoma (OSCC) constitutes a major public health burden worldwide and accounts for a disproportionately high cancer incidence in India, representing nearly one-third of all malignancies among males (1). Among the anatomical subsites of the oral cavity, the gingivo-buccal complex—including the buccal mucosa, gingivo-buccal sulcus, and alveolar ridge—is the most frequently affected subsite in the Indian population due to the widespread habit of smokeless tobacco consumption (2, 3). Chronic exposure to tobacco quid, and commercially available chewing tobacco preparations contributes significantly to persistent mucosal injury, inflammation, and malignant transformation in this subsite (4).

Despite advances in surgery, radiotherapy, and systemic therapy, the prognosis of gingivo-buccal OSCC (GB-OSCC) remains poor because of the majority of patients presenting with locally advanced disease characterized by extensive local invasion, nodal metastasis, and aggressive tumor biology (5). Despite the histopathological parameters such as depth of invasion, invasive tumor front (ITF) characteristics, lymphovascular invasion, and tumor budding being the established indicators of disease aggressiveness (6); reliable molecular biomarkers capable of predicting tumor behavior and treatment outcomes often remain limited (7). Importantly, the biological heterogeneity of the disease observed across different oral cavity subsites suggests that subsite-specific molecular characterization is important to provide clinically relevant prognostic information than generalized OSCC profiling (8, 9).

Proteomics has emerged as a powerful high-throughput strategy for identifying dysregulated proteins involved in tumor initiation, progression, invasion, and therapeutic resistance. Unlike genomic analyses, proteomic approaches provide insights into functional molecular alterations occurring at the protein level, enabling a more direct understanding of disease biology. Several studies have explored proteomic alterations in OSCC using technologies such as two-dimensional gel electrophoresis (2DE), matrix-assisted laser desorption/ionization-time of flight (MALDI-TOF) mass spectrometry, tandem mass tag-based quantitative proteomics, and laser microdissection-assisted profiling. These studies have identified proteins associated with cytoskeletal remodeling, metabolic reprogramming, epithelial differentiation, angiogenesis, and inflammatory signaling in oral carcinogenesis (10).

Given the unique epidemiological and etiological characteristics of gingivo-buccal cancers in India, identification of validated subsite-specific biomarkers may improve risk stratification and enhance translational applicability in routine pathology practice. The previous study demonstrated distinct proteomic alterations in oral tongue squamous cell carcinoma, including the prognostic significance of vimentin expression (11). Additional studies have identified proteins such as CSTB, NDRG1, PGK1, ITGAV, and COL6A1 as markers associated with tumor invasion and disease progression in OSCC.

This study employed an integrated discovery-to-validation workflow using proteomic profiling and immunohistochemistry to identify proteins associated with disease progression and aggressive clinicopathological behavior in GB-OSCC. Differentially expressed proteins identified by 2DE and MALDI-TOF mass spectrometry were subjected to bioinformatic pathway analysis, followed by validation of selected candidate biomarkers—Carbonic Anhydrase 1 (CA1), Hemoglobin subunit beta (HBB), Torsin A, and *α*-Tubulin—in healthy controls, oral epithelial dysplasia, and GB-OSCC tissues. Further the study evaluated their association with histopathological indicators of tumor aggressiveness and disease progression.

However, most of the published studies have focused on heterogeneous oral cavity cohorts without emphasizing the gingivo-buccal subsite, and only a limited number of candidate biomarkers have undergone systematic clinicopathological validation in Indian populations with tobacco-associated disease (12, 13). Despite sustained efforts from researchers world-wide, there is currently no site-specific protein-based biomarkers, especially for the Indian population, where the disease burden is high. A systematic review published on proteomic expression profiling in OSCC, has expressed the lack of validation studies on the numerous protein-based biomarkers identified from the respective studies and the need for the same (14). Candidate proteins for immunohistochemical (IHC) validation were selected using predefined criteria that included magnitude of differential expression, consistency of expression across the study groups, biological relevance to tumor progression, novelty in gingivo-buccal OSCC, and suitability for IHC validation on formalin-fixed paraffin-embedded (FFPE) tissues.

The primary objective of this study was to identify differentially expressed proteins in gingivo-buccal OSCC that correlated with clinical outcomes using a proteomic approach. Secondary objective was to validate selected proteins (CA1, HBB, Torsin A, and *α*-Tubulin) across the spectrum of oral carcinogenesis from normal mucosa and dysplasia to squamous cell carcinoma to evaluate their potential as diagnostic and prognostic biomarkers. HBB, Torsin A, CA1, and *α*-Tubulin were selected for validation using a combination of quantitative expression characteristics and biological rationale. HBB showed the highest normalized abundance among proteins detected exclusively in the early-stage failure group (Group 4; normalized abundance 2.73258). Among proteins detected in more than one clinical group, Torsin A showed the largest fold difference (19.7394), followed by CA1 (4.88747) and *α*-Tubulin/TUBA1A (4.44467) (Table 1).

**Table 1:** Proteins identified by mass spectrometry analysis in Group 1 (late-stage failure), Group 2 (late-stage NED), Group 3 (early-stage NED), and Group 4 (Early-stage failure).

Match ID	Max	Fold	Match count	Group 1	Group 2	Group 3	Group 4	Accession no	Protein name
1	4.50316	2.07435	2	—	—	4.50316	2.17088	—	—
4	2.53044	1.3045	2	—	—	2.53044	1.93977	—	—
*5	**2.73258**	**—**	**1**	**—**	**—**	**—**	**2.73258**	**HBB_HUMAN**	**Hemoglobin subunit beta**
6	2.23714	—	1	—	—	—	2.23714	Q5NV90_HUMAN	V2-17 protein (Fragment)
7	1.35849	—	1	—	—	—	1.35849	—	—
8	0.849261	—	1	—	—	—	0.849261	ROP1A_HUMAN	Ropporin-1A
9	1.7641	—	1	—	—	—	1.7641	RAB37_HUMAN	Ras-related protein Rab-37
10	1.14759	—	1	—	—	—	1.14759	HSPB1_HUMAN	Heat-shock protein beta-1
11	1.44433	—	1	—	—	—	1.44433	—	—
*15	**9.44284**	**5.03154**	**3**	**9.44284**	**1.87673**	**4.6382**	**—**	**CAH1_HUMAN**	**Carbonic anhydrase 1**
*16	**12.5698**	**4.88747**	**4**	**5.27918**	**2.57184**	**12.5698**	**3.06997**	**CAH1_HUMAN**	**Carbonic anhydrase 1**
17	5.83185	2.21431	3	3.71637	—	5.83185	2.63371	CAH3_HUMAN	Carbonic anhydrase 3
18	1.82134	—	1	—	—	—	1.82134	CAH2_HUMAN	Carbonic anhydrase 2
19	2.07398	2.6394	2	—	—	2.07398	0.785777	—	—
20	3.58028	2.71262	2	—	—	3.58028	1.31986	B4DUJ3_HUMAN	cDNA FLJ52895, highly similar to carbonic anhydrase 3 (EC 4.2.1.1)
22	7.28042	10.6545	3	7.28042	5.42438	—	0.68332	—	—
25	3.02792	—	1	—	—	—	3.02792	G3P_HUMAN	Glyceraldehyde-3-phosphate dehydrogenase
26	2.96605	1.55897	2	—	—	2.96605	1.90258	—	—
27	1.98242	1.68921	2	—	—	1.98242	1.17358	E7EUT5_HUMAN	Glyceraldehyde-3-phosphate dehydrogenase
*28	**0.8946**	**19.7394**	**2**	**—**	**—**	**0.04532**	**0.8946**	**TOR1A_HUMAN**	**Torsin-1A**
29	0.236962	—	1	—	—	—	0.236962	BGH3_HUMAN	Transforming growth factor-beta-induced protein ig-h3
31	0.420623	—	1	—	—	—	0.420623	—	—
33	0.552722	—	1	—	—	—	0.552722	Q86VG2_HUMAN	Splicing factor proline/glutamine-rich (Polypyrimidine tract binding protein associated)
34	1.35677	—	1	—	—	-	1.35677	K1C14_HUMAN	Keratin, type I cytoskeletal 14
38	0.887975	—	1	—	—	—	0.887975	K2C6A_HUMAN	Keratin, type II cytoskeletal 6A
40	1.09989	—	1	—	—	—	1.09989	K1C16_HUMAN	Keratin, type I cytoskeletal 16
41	1.67431	—	1	—	—	—	1.67431	E7EUT5_HUMAN	Glyceraldehyde-3-phosphate dehydrogenase
43	0.905799	—	1	—	—	—	0.905799	—	—
44	0.465701	5.40777	2	—	—	0.086117	0.465701	ZN525_HUMAN	Zinc-finger protein 525
45	0.311425	6.58608	2	—	—	0.047285	0.311425	A8K0L8_HUMAN	cDNA FLJ76998, highly similar to *Homo sapiens* zinc finger protein 28 homolog (mouse), mRNA
46	0.581649	1.19565	2	—	—	0.581649	0.486469	—	—
47	0.900794	1.12541	2	—	—	0.800413	0.900794	—	—
48	0.659749	—	1	—	—	—	0.659749	H0YA55_HUMAN	Serum albumin (fragment)
49	1.10951	—	1	—	—	—	1.10951	ALBU_HUMAN	Serum albumin
50	1.11365	—	1	—	—	—	1.11365	—	—
*52	**0.39756**	**—**	**1**	**—**	**—**	**—**	**0.39756**	**TBA4A_HUMAN**	**Tubulin alpha-4A chain**
54	0.438857	—	1	—	—	—	0.438857	K2C5_HUMAN	Keratin, type II cytoskeletal 5
55	0.342179	—	1	—	—	—	0.342179	PRDX2_HUMAN	Peroxiredoxin-2
60	0.204298	—	1	—	—	—	0.204298	IF1AY_HUMAN	Eukaryotic translation initiation factor 1A, Y-chromosomal
62	0.672245	—	1	—	—	—	0.672245	IF2A_HUMAN	Eukaryotic translation initiation factor 2 subunit 1
63	2.48625	1.21317	2	2.04938	—	2.48625	—	KRT2	Keratin, type II cytoskeletal 2 epidermal
64	1.88579	8.5447	3	1.88579	—	1.73209	0.220697	—	—
65	1.85758	1.65475	3	1.70283	—	1.85758	1.12257	K2C6B_HUMAN	Keratin, type II cytoskeletal 6B
67	4.21765	1.37207	2	4.21765	3.07393	—	—	—	—
68	6.52478	1.83401	2	3.55766	6.52478	—	—	TPI1_HUMAN	Triosephosphate isomerase
71	2.92249	1.83193	2	2.92249	1.59531	—	—	—	—
72	0.331303	—	1	0.331303	—	—	—	—	—
73	1.4024	—	1	1.4024	—	—	—	—	—
74	4.35353	—	1	4.35353	—	—	—	DBI	Diazepam binding inhibitor,
75	1.99961	—	1	—	1.99961	—	—	—	—
76	0.791514	1.09458	2	0.723124	0.791514	—	—	—	—
77	1.29562	1.17601	2	1.10171	1.29562	—	—	—	—
*78	**2.39375**	**4.44467**	**2**	**0.538566**	**2.39375**	**—**	**—**	**TUBA1A**	Tubulin alpha-1A chain
79	2.25033	1.74222	2	1.29165	2.25033	—	—	—	—
80	2.81639	5.62276	2	0.500891	2.81639	—	—	—	cDNA FLJ55253, highly similar to Actin, cytoplasmic 1

*Proteins validated by immunohistochemistry.

Protein abundance values are derived from pooled tissue samples and are presented to facilitate candidate biomarker selection. No patient-level statistical inference was performed from the discovery proteomic dataset.

## 2 Materials and methods

### 2.1 Study design and patient cohort

This study employed a two-phase discovery and validation design to identify and validate protein biomarkers associated with gingivo-buccal oral squamous cell carcinoma (GB-OSCC). The study was approved by the respective Institutional Ethics Committees of Sree Balaji Dental College and Hospital, Chennai (IEC No. SBDCH/2017/OP/1102), Cancer Institute (WIA), Chennai (CIWIA/Protocol 1/HNCOG 2014), and Sri Ramachandra Institute of Higher Education and Research, Chennai (IEC No. 19/DEC/72/118). All procedures were performed in accordance with the Declaration of Helsinki, and written informed consent was obtained from all participants prior to sample collection.

The discovery phase included prospectively collected fresh-frozen tissue specimens from 40 histopathologically confirmed GB-OSCC patients recruited between December 2018 and January 2022. Clinical data including demographic characteristics, tobacco exposure, alcohol consumption, and treatment outcomes were recorded for all patients. Patients were categorized into four clinically relevant groups based on tumor stage and follow-up outcome status: advanced-stage failure (Stage III/IV; *n* = 10), advanced-stage no evidence of disease (NED; *n* = 10), early-stage NED (Stage I/II; *n* = 10), and early-stage failure (*n* = 10). Clinical outcome classification was based on a minimum follow-up duration of 2 years (Supplementary Table S1).

Fresh tumor tissues were snap-frozen immediately after biopsy confirmation and stored in liquid nitrogen until protein extraction. Due to limited protein yield from individual samples, tissues within each clinical group were pooled prior to proteomic analysis to obtain representative protein expression profiles while minimizing inter-sample technical variability.

The pooled samples represented one biological sample for each clinical group. Duplicate 2DE runs were performed as technical replicates to assess the reproducibility of protein separation and spot detection. As pooling precludes estimation of inter-patient biological variability, the proteomic discovery phase was designed as an exploratory analysis for candidate biomarker image analysis identified more than 75 protein spots, among which 37 proteins demonstrated significant differential upregulation between the study groups identification rather than for patient-level statistical inference.

To reduce etiological heterogeneity, only patients with tobacco-chewing habits were included in the discovery cohort. The validation phase consisted of retrospective FFPE tissue specimens (*n* = 125) obtained from patients diagnosed with gingivo-buccal dysplasia or GB-OSCC. Healthy control tissues were obtained from gingivo-buccal mucosa collected during surgical extraction of impacted third molars following informed consent. Hematoxylin and eosin-stained sections were independently reviewed by two oral pathologists to confirm histopathological diagnosis and tumor grading.

Histopathological evaluation included assessment of ITF characteristics using Bryne’s grading system, including degree of keratinization, nuclear pleomorphism, invasion pattern, and lymphoplasmacytic infiltrate (15). Tumor depth of invasion was measured using ProgRes CapturePro v2.8.8 software (JENOPTIK, Germany) at 4× magnification from the basement membrane to the deepest point of invasion, as previously described (16). During antigen retrieval, a small number of tissue sections were lost, resulting in final evaluable sample numbers of CA1 (*n* = 107), HBB (*n* = 107), Torsin A (*n* = 106) and *α*-Tubulin (*n* = 106).

### 2.2 Protein extraction

Pooled frozen tissue samples from each clinical group were pulverized under liquid nitrogen using a pre-chilled mortar and pestle. Proteins were extracted using lysis buffer containing 7 M urea, 2 M thiourea, 4% CHAPS, 20 mM phenylmethylsulfonyl fluoride, and 20 mM dithiothreitol. Tissue lysates were sonicated for 10 min and centrifuged at 12,000 rpm for 15 min at 4 °C (17). Protein concentrations were quantified using the Bradford assay, and aliquots were stored at −80 °C until further analysis.

### 2.3 Two-dimensional gel electrophoresis

2DE was performed for comparative proteomic profiling (18). Briefly, 13 cm immobilized pH gradient (IPG) strips (pH 3–10; GE Healthcare, Sweden) were rehydrated and subjected to isoelectric focusing using an IPGPhor III system (GE Healthcare, Sweden). The focusing conditions included sequential voltage gradients of 100 V for 1 h, 300 V for 2 h, 1,000 V for 1 h, and 5,000 V for 5 h, followed by a final step-and-hold phase at 5,000 V until a total of 50,000 Vh was achieved at 20 °C. Following isoelectric focusing, IPG strips were equilibrated sequentially in buffers containing dithiothreitol and iodoacetamide. Second-dimension separation was performed using 12.5% SDS-PAGE in an SE600 electrophoresis system (GE Healthcare, Sweden) at 1 W/gel for 1 h followed by 13 W/gel for 3 h.

### 2.4 In-gel digestion and MALDI-TOF mass spectrometry

Protein spots were visualized using colloidal Coomassie Brilliant Blue G-250 staining and scanned using a high-resolution scanner (ScanMaker 9700XL, Microtek) (19). Differentially expressed protein spots were analyzed using PDQuest software version 8.01 (Bio-Rad, USA). Selected protein spots were excised and subjected to in-gel trypsin digestion. Gel fragments were destained using 50% acetonitrile and 25% ammonium bicarbonate, dehydrated in 100% acetonitrile, and vacuum-dried. Digestion was performed overnight at 37 °C using sequencing-grade trypsin (Sigma-Aldrich, USA). Peptides were extracted using trifluoroacetic acid/acetonitrile solutions and concentrated by vacuum drying (20–22).

Peptide mass fingerprinting was performed using MALDI-TOF mass spectrometry with external calibration in the 700–4,000 Da range. Spectral analysis was conducted using FlexAnalysis software version 2.4 (Bruker Daltonics, Germany), and protein identification was performed using the Mascot search engine version 2.2 against the NCBInr database (11, 23). Protein identification was accepted only when Mascot scores exceeded the significance threshold and the peptide mass fingerprint demonstrated acceptable sequence coverage. Protein identification parameters are summarized in Supplementary Table S2.

### 2.5 Functional enrichment and protein interaction analysis

Differentially expressed proteins identified by MALDI-TOF analysis were subjected to functional enrichment analysis using the PANTHER database. Protein–protein interaction (PPI) networks were generated using the STRING database to evaluate functional associations and pathway enrichment. Interaction networks were analyzed based on experimentally validated interactions, co-expression, genetic context, and curated biological knowledge to identify pathways associated with tumor progression and aggressive disease behavior (24).

### 2.6 Immunohistochemistry

IHC validation was performed on 4 μm FFPE tissue sections mounted on 3-aminopropyltriethoxysilane-coated slides. Sections were deparaffinized, rehydrated, and subjected to endogenous peroxidase blocking using hydrogen peroxide. Antigen retrieval was performed using 0.05 M Tris-EDTA buffer (pH 8.5) in a pressure cooker. Sections were incubated overnight at 4 °C with primary antibodies against Torsin A (Santa Cruz Biotechnology; 1:50), *α*-Tubulin (Santa Cruz Biotechnology; 1:150), Carbonic Anhydrase 1 (Santa Cruz Biotechnology; 1:50), and Hemoglobin *β* (Santa Cruz Biotechnology; 1:100). Detection was performed using the Polyexcel HRP/DAB detection system (PathnSitu, USA), followed by hematoxylin counterstaining.

IHC expression was evaluated using a semi-quantitative scoring system, in which both the percentage of positively stained tumor cells and staining intensity were assessed to generate an immunoreactivity score (H-score). CA1 IHC positivity was graded as percentage positivity. The staining intensity was recorded as mild, moderate, and intense. The % positivity was further scored as −0 (negative), score 1(< 20%), score 2 (> 20%). Final H score was calculated by multiplying the % positivity score with intensity. The score of 0 and 1 was considered as negative and > 1 was considered positive (25). Similarly, HBB % score was graded as − negative (< 1%), score I (1–10%), score II (10–30%), score III (30–60%), and score IV (> 60%). The intensity of stain was noted as mild (1), moderate (2), and intense (3). Final H score was obtained by multiplying the % score with intensity and H score was graded as 0 = (negative), grade 1- (1–3), grade 2- (4–6), grade 3- (>6) (26, 27). The HBB localization was seen in either the cytoplasm or cytoplasmic membranes. The percentage positivity of Torsin A and *α*-Tubulin was scored as −0 = (negative), score 1- (1–10%), score 2 − (11–50%), score 3- > 50%. The intensity of stain was noted as mild (1), moderate, (2) and intense (3). Semi-quantitative H score was obtained by multiplying the %score with intensity and H score was graded as 0- negative, grade 1- (1–3), grade 2- (4–6), grade 3- (>6) (28–30).

IHC scoring was independently performed by two blinded oral pathologists. The pathologists were blinded to all clinical and pathological information. IHC grading was performed by both pathologists through direct visualization of the slides on a computer screen. Although the sections were reviewed simultaneously, the scoring was performed independently by each pathologist. Any discrepancies in scoring were re-evaluated jointly on the same day, and a consensus score was assigned. Staining intensity and percentage positivity were evaluated across 10 high-power fields (40× magnification). Interobserver agreement was excellent (*κ* = 1.0). Any discrepancies were resolved by joint review and consensus.

### 2.7 Statistical analysis

Statistical analyses were performed using SPSS software version 16.0 (IBM Corp., USA) (31). Student’s *t*-test was used to assess differences in protein abundance during proteomic analysis. ROC curve analysis was performed to evaluate the diagnostic performance of each biomarker in distinguishing gingivo-buccal squamous cell carcinoma from non-malignant tissues. The thresholds used for ROC analysis were selected based on the semi-quantitative IHC scoring framework employed in this study and were applied as operational thresholds for exploratory analysis. Specifically, IHC positivity of >20% positive cells were used for CA1, whereas >10% positive cells were used for HBB, Torsin A, and *α*-Tubulin. These thresholds were not determined by maximizing the Youden index or by other ROC-based optimization methods. Associations between biomarker expression and clinicopathological variables were analyzed using the chi-square test. As multiple clinicopathological variables were evaluated for each biomarker, no formal correction for multiple comparisons was applied. Accordingly, the reported *p* values represent nominal significance levels and should be interpreted as exploratory.

## 3 Results

### 3.1 Proteomic profiling identified differentially expressed proteins associated with disease progression in gingivo-buccal OSCC

**Figure 1:**
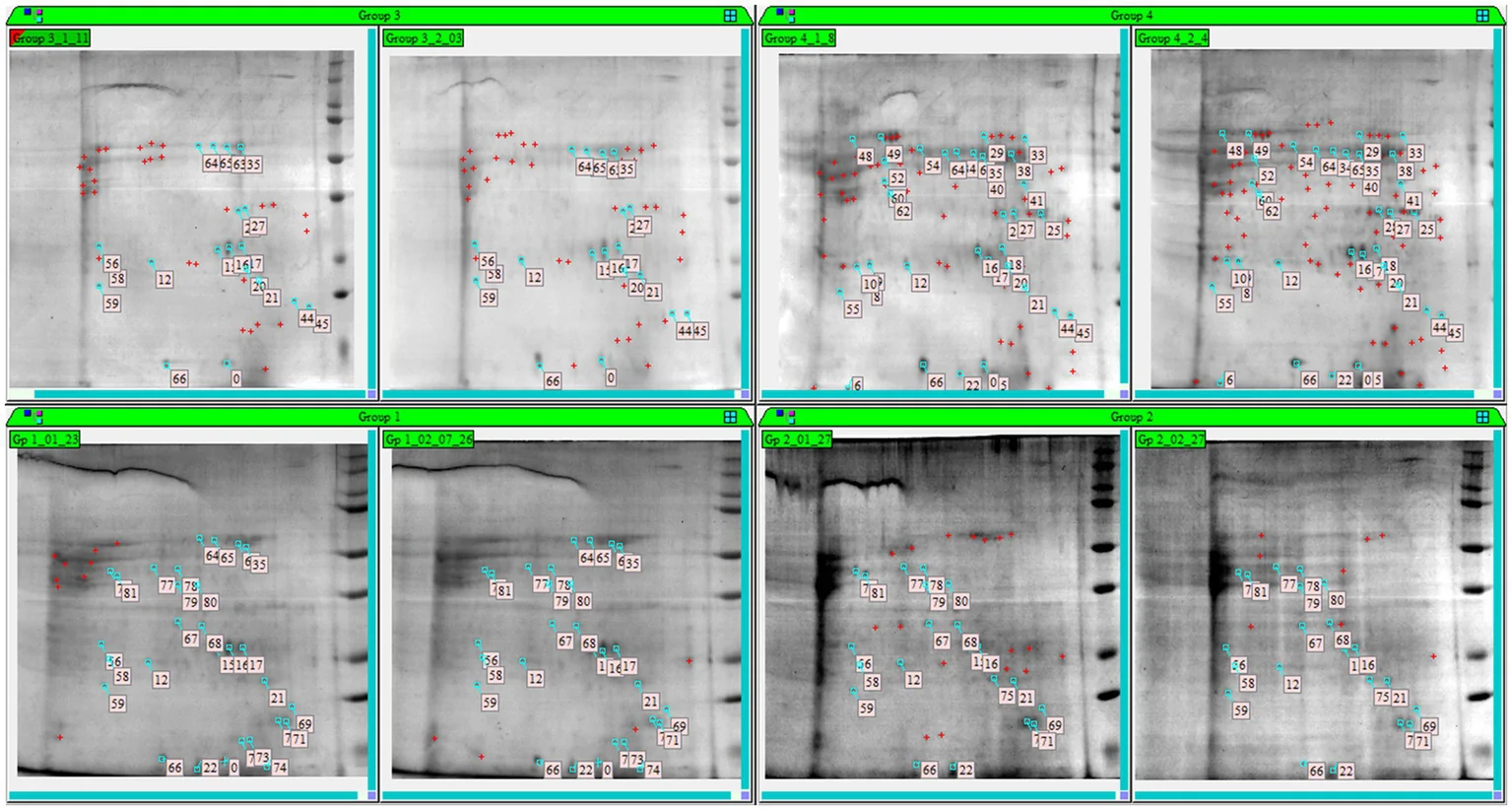
Representative two-dimensional gel electrophoresis (2DE) gel images of four study groups showing differentially expressed protein spots selected for MALDI-TOF/MS identification.

Comparative proteomic profiling was performed using pooled fresh-frozen tissue samples obtained from 40 histopathologically confirmed GB-OSCC cases categorized into four clinically defined groups: advanced-stage failure, advanced-stage NED, early-stage NED, and early-stage failure. Representative two-dimensional electrophoresis (2DE) gel images demonstrated distinct protein expression patterns across the four clinical groups (Figure 1).

Image analysis identified more than 75 protein spots, among which 37 proteins showed differential expression across the pooled clinical groups. Because the discovery phase was performed using pooled tissue samples, these findings should be interpreted as exploratory and descriptive rather than as patient-level statistical comparisons. The differentially expressed proteins were subsequently prioritized for validation in an independent IHC cohort consisting of individual patient samples. The identified proteins included cytoskeletal proteins, metabolic enzymes, stress–response proteins, and signaling-associated molecules implicated in tumor progression and invasion (Table 1; Supplementary Table S2). Protein identification was carried out using the Mascot search engine against the NCBInr database. Protein assignments were considered reliable only if they fulfilled the following criteria: (i) identification by at least two unique peptides, (ii) peptide confidence of ≥95%, and (iii) protein confidence of ≥99%. A target-decoy strategy was employed to control the false discovery rate (FDR), which was maintained below 1% at both the peptide and protein levels. Sequence coverage and Mascot protein scores for each identified protein were extracted from the Mascot output files.

Among the differentially expressed proteins, CA1, HBB, TOR1A, and *α*-Tubulin were selected for further validation based on their differential expression patterns, biological relevance, and unique distribution across the clinical groups. HBB and Tubulin alpha-4A were predominantly identified in the early-stage failure group, whereas Torsin A was enriched in early-stage disease groups. CA1 expression was consistently observed across all clinical categories, suggesting its potential role in both tumor initiation and progression.

### 3.2 Functional enrichment and protein interaction network analysis

**Figure 2:**
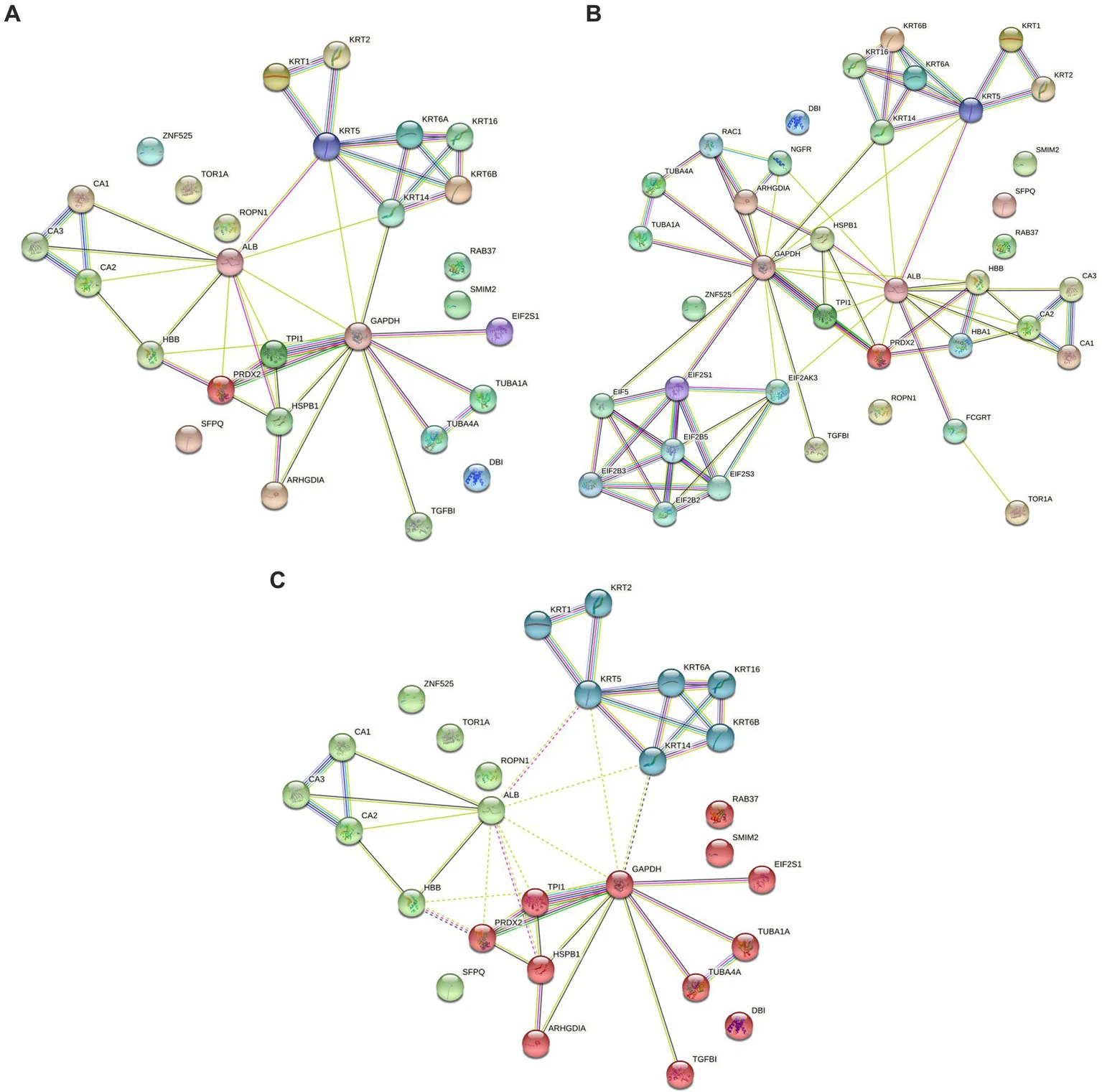
**(A)** Protein–protein interaction network of upregulated proteins identified by MALDI-TOF/MS constructed using the STRING database. **(B)** Expanded String Network of upregulated proteins. identified by MALDI-TOF/MS. **(C)** STRING protein–protein interaction network highlighting functional clusters among upregulated proteins.

PPI analysis using the STRING database revealed extensive functional connectivity among the differentially expressed proteins (Figures 2A–C). Seven proteins, including ZNF525, TOR1A, ROPN1, SFPQ, RAB37, SMIM2, and DBI, appeared as isolated nodes without documented direct interactions with the remaining proteins in the dataset. In contrast, the majority of proteins formed interconnected functional networks associated with metabolic regulation, cytoskeletal remodeling, and stress–response signaling.

Cluster analysis identified three major protein interaction clusters. The first cluster predominantly consisted of carbonic anhydrase family proteins and cytosolic metabolic regulators, while the second cluster included keratin-associated cytoskeletal proteins. The third cluster comprised proteins involved in oxidative stress response, heat-shock signaling, and tubulin-associated pathways.

Functional annotation using the PANTHER database demonstrated that the majority of the identified proteins possessed binding and catalytic functions and were primarily associated with cellular and metabolic processes (Supplementary Figures S1A–C). Pathway enrichment analysis further revealed involvement of these proteins in angiogenesis, glycolysis, apoptosis signaling, and CCKR signaling pathways (Supplementary Figures S2A,B), highlighting their potential contribution to tumor progression and aggressive disease biology in GB-OSCC.

### 3.3 IHC validation of candidate biomarkers

IHC validation was performed in an independent cohort comprising healthy controls, oral epithelial dysplasia, and GB-OSCC tissue samples (*n* = 125). Following exclusion of tissue sections lost during antigen retrieval, the final evaluable cohort included CA1 (*n* = 107), HBB (*n* = 107), Torsin A (*n* = 106), and *α*-Tubulin (*n* = 106).

### 3.4 CA1 expression correlates with aggressive pathological features

CA1 expression was detected in 72% (77/107) of evaluable tissues and demonstrated significantly increased expression in GB-OSCC compared with healthy controls and dysplastic lesions. Increased CA1 expression was significantly associated with age (*p* < 0.002) tobacco exposure, including both tobacco chewing (*p* < 0.010) duration (*p* < 0.012) and smoking history (*p* < 0.000), alcohol consumption (*p* < 0.001) and indulging in multiple habits like chewing tobacco, smoking, and alcohol (*p* < 0.000) (Supplementary Table S3).

**Figure 3:**
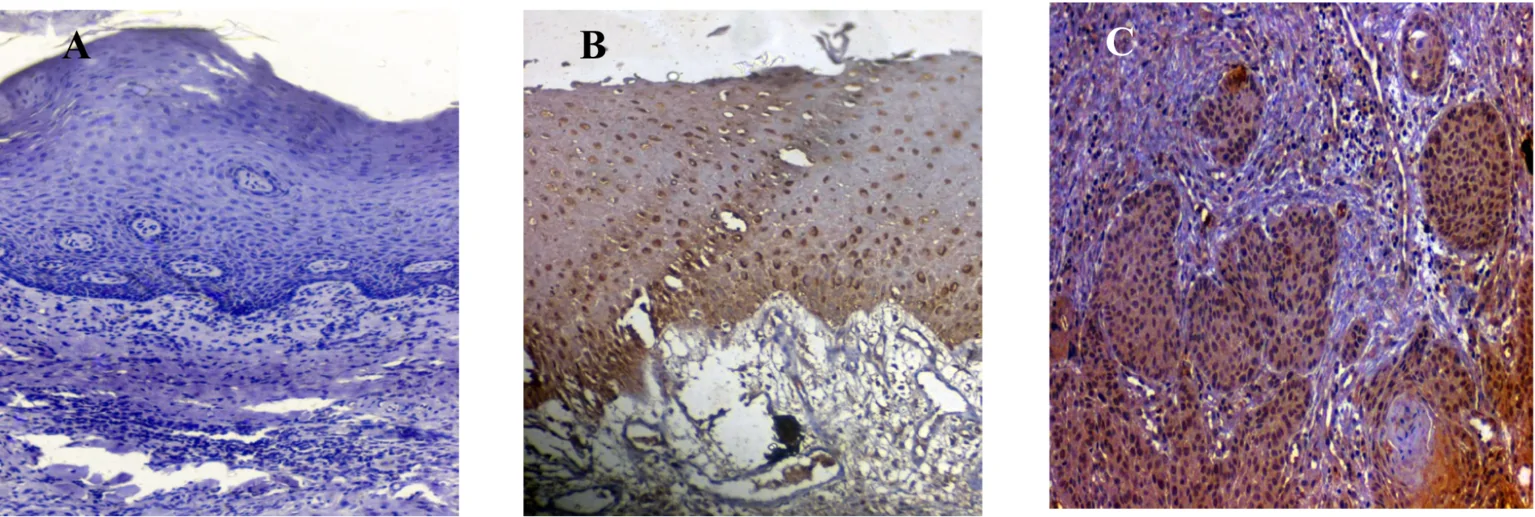
**(A)** Normal oral epithelium showing negative stain for CA1, IHC, 20×. **(B)** Moderate dysplastic epithelium with intense nuclear and cytoplasmic positivity for CA1, IHC, 20×. **(C)** MDSCC with intense cytoplasmic and nuclear positivity for CA1, IHC, 20×.

Clinicopathological analysis demonstrated significant correlations between CA1 expression and tumor differentiation (*p* < 0.001), tumor size (*p* < 0.001), muscle invasion (*p* < 0.001), keratinization at the ITF (*p* < 0.001), invasive growth pattern (*p* < 0.001), Bryne’s grading score (*p* < 0.001), depth of invasion (*p* < 0.001), lymphovascular invasion (*p* < 0.001), and tumor budding (*p* < 0.001) (Table 2). Increased CA1 expression was also associated with intense stromal inflammatory infiltrates. Immunohistochemically, CA1 expression demonstrated progressive enhancement from weak or absent staining in healthy oral epithelium to strong cytoplasmic and nuclear positivity in dysplastic epithelium and invasive carcinoma (Figures 3A–C).

**Table 2:** Receiver operating characteristic curve analysis of CA1, HBB, Torsin A, and *α*-Tubulin for the diagnosis of gingivo-buccal squamous cell carcinoma.

Biomarker	AUC	Std error	*p*-value	95% CI (lower–upper)
CA1	0.712	0.053	0.000	0.608–0.815
HBB	0.769	0.048	0.000	0.675–0.863
Torsin A	0.634	0.058	0.020	0.520–0.748
*α*-Tubulin	0.639	0.058	0.017	0.525–0.753

### 3.5 HBB expression associated with tumor aggressiveness and invasive behavior

HBB expression was observed in 62.6% (67/107) of analyzed samples and demonstrated significantly higher expression in moderately differentiated squamous cell carcinoma compared with healthy oral mucosa and dysplastic lesions. Although no significant associations were identified with demographic variables or tobacco-related habits, HBB expression showed strong correlations with tumor progression parameters.

**Figure 4:**
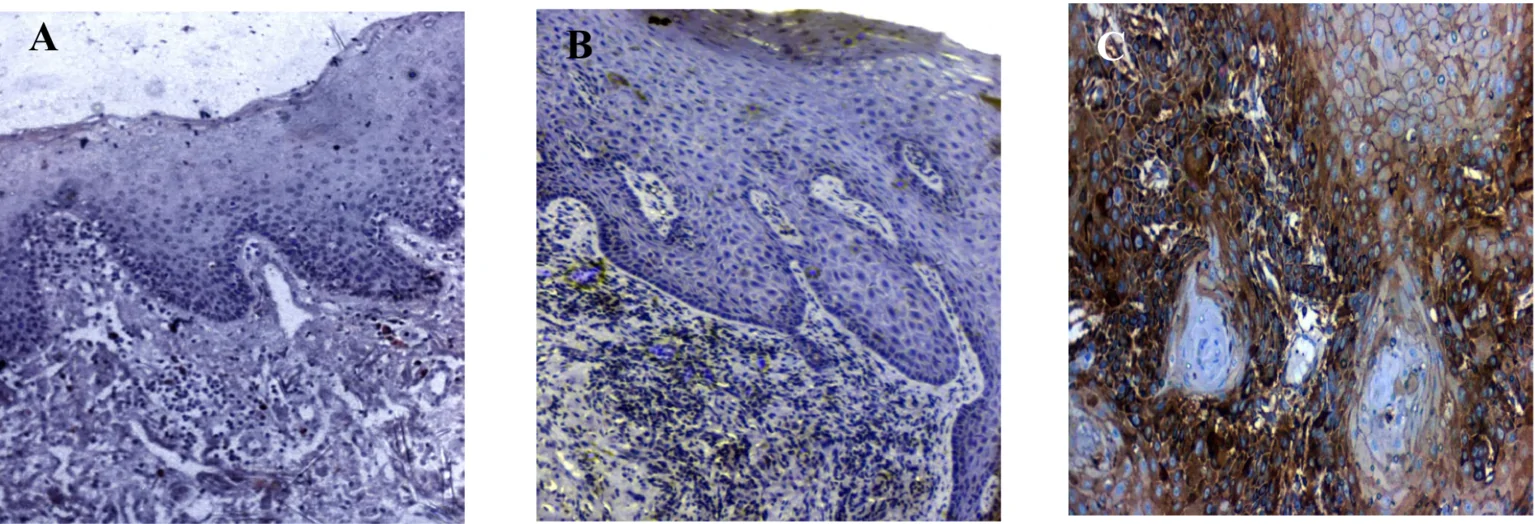
**(A)** Normal oral epithelium showing negative stain for Hemoglobin *β* Antibody, IHC, 20×. **(B)** Moderate dysplasia with complete negative staining for Hemoglobin *β* Antibody IHC, 20×. **(C)** WDSCC with intense cytoplasmic and cell membrane positivity for Hemoglobin *β* Antibody, IHC, 20×.

Significant associations were identified between HBB expression and tumor size (*p* < 0.001), inflammatory infiltrate (*p* = 0.032), muscle invasion (*p* < 0.001), degree of keratinization at ITF (*p* < 0.001), pattern of invasion at ITF (*p* < 0.001), Bryne’s grade at ITF (*p* < 0.001), depth of invasion (*p* < 0.001), lymphovascular invasion (*p* < 0.001), and tumor budding (*p* < 0.001) (Supplementary Table S4). IHC analysis demonstrated predominantly cytoplasmic and membranous staining in invasive carcinoma tissues, whereas healthy mucosa and dysplastic lesions showed absent or weak expression (Figures 4A–C).

### 3.6 Torsin A overexpression associated with advanced histopathological features

Torsin A expression was identified in 89.6% (95/107) of tissues analyzed and demonstrated significantly increased expression in both well-differentiated and moderately differentiated squamous cell carcinoma compared with healthy controls and dysplastic lesions.

**Figure 5:**
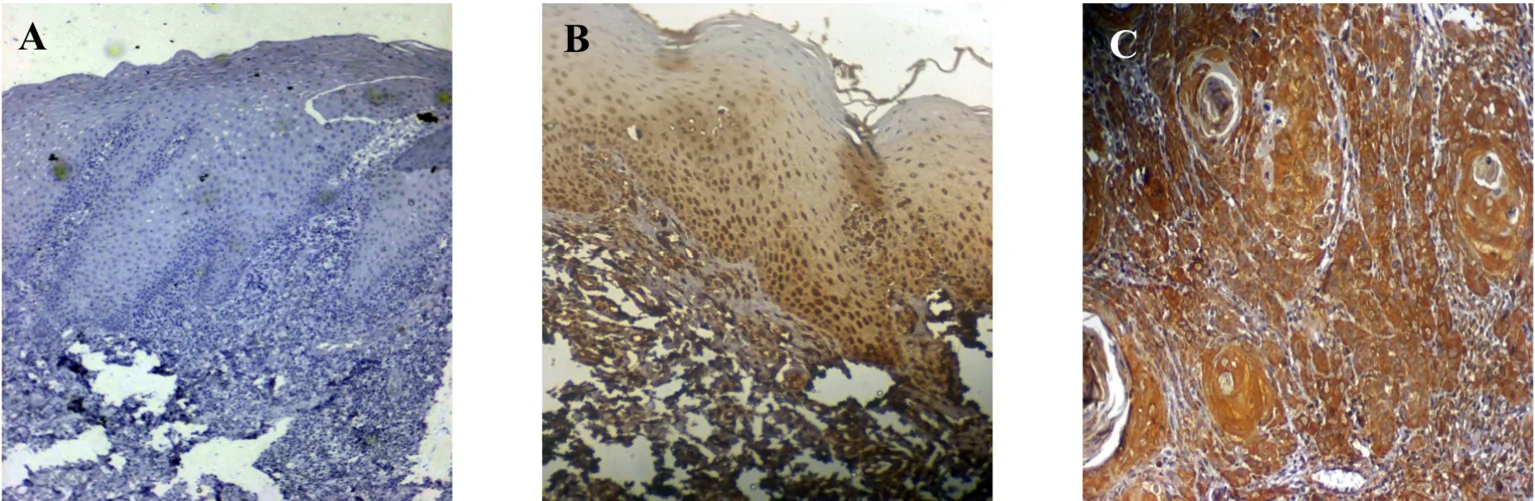
**(A)** Normal oral hyperplastic epithelium showing negative stain for Torsin A, IHC, 20×. **(B)** Moderate dysplasia with intense nuclear and cytoplasmic positivity for Torsin A, IHC, 20×. **(C)** WDSCC with intense cytoplasmic and nuclear positivity for Torsin A, IHC, 20×.

Torsin A expression showed significant associations with tumor size (*p* = 0.012), muscle invasion (*p* < 0.001), degree of keratinization at ITF (*p* < 0.001), invasion pattern at ITF (*p* = 0.001), Bryne’s grade at ITF (*p* < 0.001), depth of invasion (*p* = 0.004), lymphovascular invasion (*p* = 0.001), and tumor budding (*p* = 0.001) (Supplementary Table S5). Strong nuclear and cytoplasmic staining was predominantly observed in invasive carcinoma tissues, whereas healthy controls and dysplastic lesions demonstrated comparatively lower expression levels (Figures 5A–C).

### 3.7 *α*-Tubulin expression correlates with tumor invasion and aggressive histopathology

**Figure 6:**
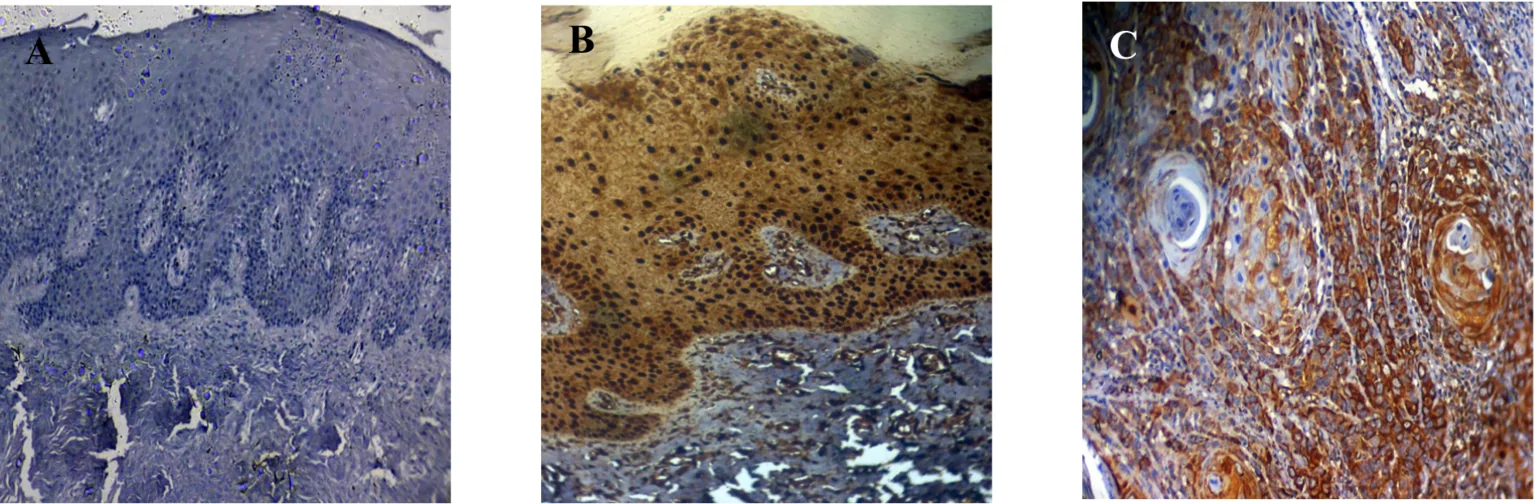
**(A)** Normal oral epithelium showing negative stain for Anti-*α* Tubulin, IHC, 20×. **(B)** Moderate dysplasia with intense nuclear and cytoplasmic positivity for anti-*α* Tubulin IHC, 20×. **(C)** WDSCC with intense cytoplasmic and cell membrane positivity for Anti-α Tubulin, IHC, 20×.

Moderate-to-intense *α*-Tubulin expression was observed in 85.8% (91/106) of evaluated samples and was significantly enriched in well-differentiated and moderately differentiated GB-OSCC compared with healthy mucosa and dysplastic lesions. No significant associations were observed with age, gender, or tobacco-related habits. However, α-Tubulin expression demonstrated significant correlations with tumor size (*p* = 0.006), muscle invasion (*p* < 0.001), degree keratinization at the ITF (*p* < 0.001), invasion pattern at ITF (*p* = 0.007), Bryne’s grade at ITF (*p* = 0.006), depth of invasion (*p* = 0.006), lymphovascular invasion (*p* < 0.001), and tumor budding (*p* < 0.001) (Supplementary Table S6). IHC analysis revealed strong cytoplasmic and membranous staining in invasive carcinoma tissues, supporting the role of *α*-Tubulin in cytoskeletal remodeling and tumor invasiveness (Figures 6A–C).

### 3.8 Receiver operating characteristic curve analysis of candidate biomarkers

**Figure 7:**
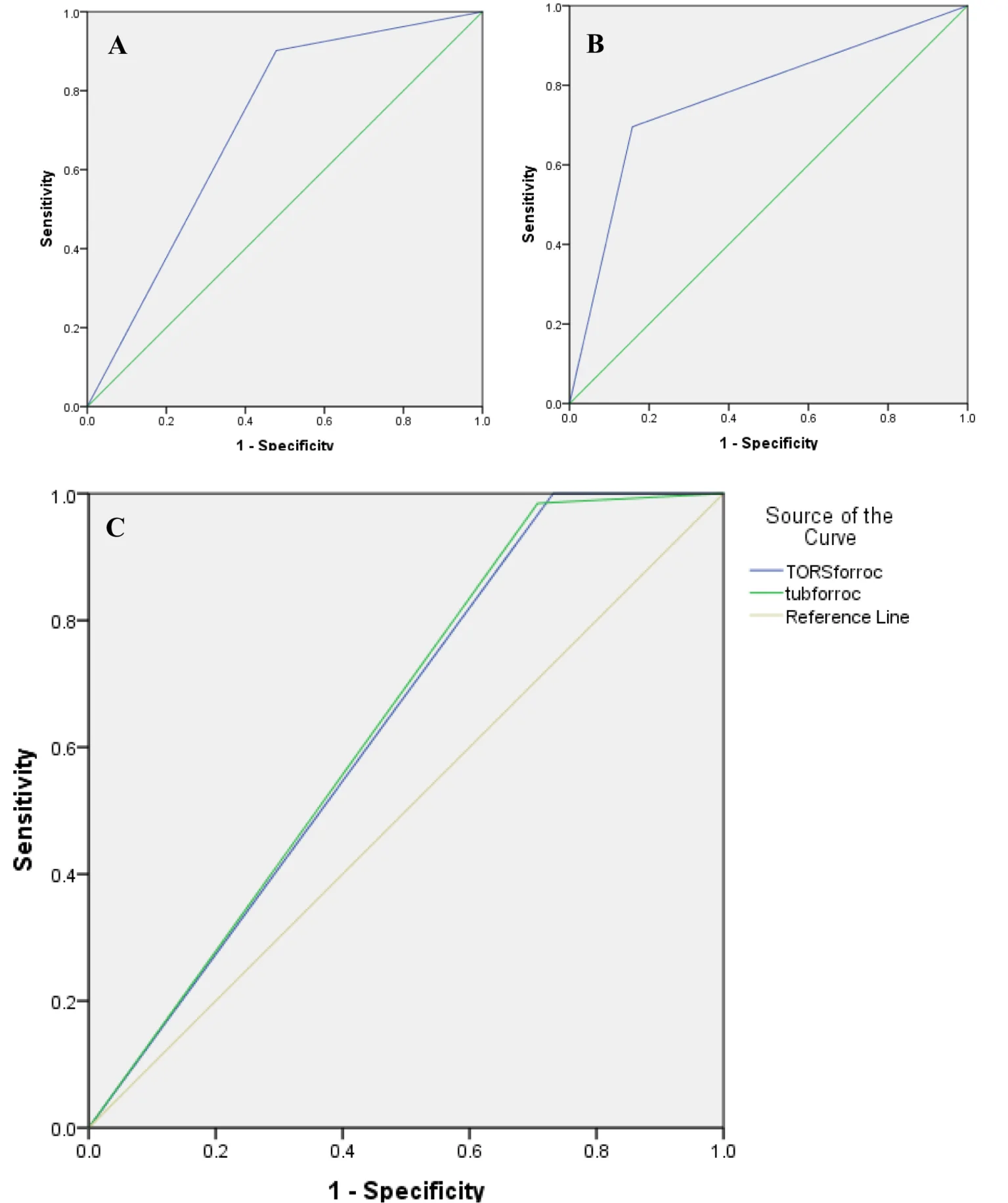
Receiver operating characteristic curve analysis of **(A)** CA1 expression, **(B)** HBB expression, and **(C)** Torsin A and *α*-Tubulin expression for the diagnosis of gingivo-buccal squamous cell carcinoma.

ROC curve analysis was performed to evaluate the ability of each biomarker to distinguish squamous cell carcinoma from non-malignant tissues (Figures 7A–C). ROC curve analysis was performed using the predefined operational thresholds described in the Methods section (>20% positive cells for CA1 and >10% positive cells for HBB, Torsin A, and *α*-Tubulin). These thresholds were selected according to the semi-quantitative IHC scoring framework used in this study and were not optimized using the present dataset.

Among the evaluated biomarkers, HBB demonstrated the highest diagnostic accuracy with an AUC of 0.769 (95% CI: 0.675–0.863; *p* < 0.001), indicating good discriminatory ability for SCC detection. CA1 also showed significant diagnostic performance with an AUC of 0.712 (95% CI: 0.608–0.815; *p* < 0.001). Torsin A and *α*-Tubulin exhibited moderate diagnostic utility with AUC values of 0.634 (95% CI: 0.520–0.748; *p* = 0.020) and 0.639 (95% CI: 0.525–0.753; *p* = 0.017), respectively. Overall, these findings suggest that the marker panel, particularly HBB and CA1, possesses potential diagnostic relevance in identifying gingivo-buccal OSCC and may aid in translational prognostic stratification (Table 2 and Figures 7A–C).

## 4 Discussion

The present study identified and validated a subsite-specific proteomic biomarker panel associated with GB-OSCC, with particular emphasis on disease aggressiveness. Using an integrated discovery-to-validation workflow combining 2DE, MALDI-TOF mass spectrometry, bioinformatic analysis, and IHC validation, four candidate proteins were identified—CA1, HBB, Torsin A, and α-Tubulin—that demonstrated progressive overexpression across the spectrum from healthy mucosa to dysplasia and invasive carcinoma. Importantly, all four proteins showed significant associations with established histopathological indicators of aggressive tumor behavior, including depth of invasion (32), ITF characteristics (6), lymphovascular invasion (33), muscle infiltration (34), and tumor budding (35). A progressive increase in biomarker expression was observed across the histopathological spectrum from healthy mucosa to dysplasia and invasive carcinoma, supporting their association with oral carcinogenesis and tumor progression.

OSCC exhibits substantial molecular heterogeneity across anatomical subsites, largely influenced by etiological exposure patterns, tumor microenvironment, and biological behavior (36). While several genomic and transcriptomic studies have explored OSCC biology, validated subsite-specific protein biomarkers for gingivo-buccal cancers remain limited, particularly in Indian populations characterized by high exposure to smokeless tobacco (37, 38). In this context, proteomics offers a functional systems-level approach that directly evaluates dysregulated protein expression and biological pathways associated with carcinogenesis and tumor progression.

During the discovery phase, tissue lysates from patients within each clinical group were pooled before proteomic analysis because of limited protein yield. Consequently, the proteomic comparisons represent group-level exploratory analyses rather than patient-level differential expression analyses. Although technical replicate gels were performed to assess reproducibility of protein separation, these do not account for biological variability between individual patients. Therefore, the proteomic findings should be interpreted as hypothesis-generating and were subsequently validated in an independent IHC cohort.

The current study identified 37 differentially expressed proteins involved in cytoskeletal organization, metabolic regulation, oxidative stress response, epithelial differentiation, and tumor-associated signaling pathways. Functional enrichment analysis demonstrated significant involvement of angiogenesis, glycolysis, apoptosis signaling, and cellular regulatory processes, supporting the metabolic and invasive reprogramming characteristic of aggressive OSCC. Several proteins identified in our study, including carbonic anhydrases (39), keratin-associated proteins, and peroxiredoxins (12), have previously been implicated in OSCC biology. However, the identification and validation of Torsin A, *α*-Tubulin isoforms, and HBB as potential biomarkers in GB-OSCC represent novel findings.

Among the validated biomarkers, *α*-Tubulin demonstrated strong associations with aggressive histopathological parameters, including ITF characteristics, muscle invasion, tumor budding, and depth of invasion. Tubulin isoforms are increasingly recognized as key regulators of cancer progression through their role in cytoskeletal remodeling, intracellular transport, epithelial–mesenchymal transition (EMT), and cancer stem cell maintenance (40, 41). Altered tubulin dynamics have been associated with increased metastatic potential, immune evasion, and therapeutic resistance across multiple solid tumors (42). While *β*-tubulin isoforms have been more extensively studied in OSCC (43, 44), the current study highlights the potential role of *α*-Tubulin isoforms, particularly TUBA4A and TUBA1A, in oral carcinogenesis. The progressive increase in α-Tubulin expression from dysplasia to invasive carcinoma suggests its potential utility as an early biomarker of malignant transformation and disease progression.

Carbonic anhydrases are well-established regulators of intracellular pH homeostasis and tumor adaptation to hypoxic microenvironments (45). Among the α-carbonic anhydrase family, CA IX has been widely investigated in OSCC as a hypoxia-associated prognostic biomarker (46). In the present study, CA1, CA2, and CA3 were identified as upregulated proteins during proteomic analysis, while CA1 demonstrated significant overexpression during IHC validation. Increased CA1 expression correlated strongly with tumor aggressiveness, including increased depth of invasion, lymphovascular invasion, tumor budding, and higher Bryne’s grade at ITF. Previous proteomic studies in oral potentially malignant disorders and OSCC have similarly reported elevated expression of carbonic anhydrases, suggesting their role in metabolic adaptation and progression toward invasive disease (39, 47, 48). The consistent expression of CA1 across both early and advanced disease groups further supports its relevance as a potential biomarker associated with oral carcinogenesis and tumor progression.

HBB emerged as another important biomarker associated with aggressive disease behavior (49). Although traditionally recognized as a component of erythrocytes, ectopic HBB expression has increasingly been reported in several malignancies, including breast, lung, and prostate cancers (50, 51). Tumor-associated HBB expression has been proposed to contribute to adaptation under hypoxic and oxidative stress conditions within the tumor microenvironment (26). In the present study, HBB expression demonstrated strong associations with muscle invasion, tumor budding, depth of invasion, and lymphovascular invasion. Notably, HBB expression progressively increased from healthy mucosa to dysplasia and invasive carcinoma, supporting its potential role in malignant transformation. While HBB has previously been reported in salivary proteomic studies of OSCC (52), the current study provides one of the first tissue-based IHC validations linking HBB expression to aggressive pathological features in GB-OSCC.

Torsin A represented one of the most novel findings of the current study. Torsin ATPases belong to the AAA+ ATPase superfamily and are involved in protein folding, membrane trafficking, and cellular stress responses (53, 54). Although Torsin A has primarily been studied in neurological disorders such as dystonia (55), its role in cancer biology remains largely unexplored. In our cohort, Torsin A demonstrated significantly increased expression in invasive carcinoma compared with dysplasia and healthy controls, with strong correlations to invasive tumor characteristics and poor histopathological prognostic indicators. These findings suggest that Torsin A may contribute to tumor progression through regulation of cellular stress adaptation, cytoskeletal dynamics, or intracellular transport pathways. Further mechanistic studies are required to elucidate its precise role in oral carcinogenesis.

Several IHC markers, including p53, Ki-67, EGFR, and podoplanin, have previously been investigated in OSCC and have shown associations with tumor proliferation, invasion, and prognosis (56, 57). However, their reported performance has varied considerably across studies, partly because of differences in tumor subsites, scoring methods, and patient populations. In contrast, the present study identified proteins involved in distinct biological pathways, including metabolic adaptation (CA1), oxidative stress (HBB), protein homeostasis (Torsin A) and cytoskeletal remodelling (*α*-Tubulin). Although direct comparison was beyond the scope of the present study, these biomarkers may provide complementary biological information and should be evaluated alongside established markers in future validation studies.

Overall, the present study identifies four candidate biomarkers associated with adverse histopathological features in gingivo-buccal OSCC.

Although promising, these findings require independent multicentric cohorts with survival and treatment-outcome data before prognostic or clinical utility can be established. A limitation of the discovery phase is that protein lysates were pooled within each clinical group before proteomic analysis because of limited protein yield from individual fresh-frozen tissues. Although duplicate 2DE gels were performed to ensure technical reproducibility, pooling precludes assessment of inter-patient biological variability and limits statistical inference at the individual patient level (58). Consequently, the proteomic analysis should be regarded as an exploratory discovery approach aimed at identifying candidate biomarkers rather than establishing statistically significant patient-level differences. The biological relevance of the selected proteins was subsequently evaluated in an independent IHC validation cohort comprising individual patient samples. Another limitation of the present study is that multiple clinicopathological associations were evaluated without formal adjustment for multiple comparisons. Consequently, the reported statistical associations should be considered exploratory and interpreted with appropriate caution. In addition, the ROC analyses were performed using operational thresholds derived from the semi-quantitative IHC scoring system rather than cut-off values optimized from the study dataset. Although this approach reduces the risk of overfitting, the diagnostic performance of these biomarkers requires confirmation in larger independent cohorts before their clinical utility can be established. Future studies should also evaluate whether a combined biomarker model provides improved diagnostic performance compared with individual biomarkers.

*Translational relevance*: The identification of a subsite-specific biomarker panel comprising CA1, HBB, Torsin A, and *α*-Tubulin may represent promising candidate biomarkers requiring prospective validation. The strong association of these proteins with invasive tumor behavior and adverse histopathological parameters suggests their possible utility in routine diagnostic pathology for risk stratification and early identification of aggressive disease. Given that immunohistochemistry is widely accessible in most tertiary care centers, this biomarker panel may serve as a feasible adjunct for prognostic assessment in tobacco-associated oral cancers, particularly in resource-limited settings with a high disease burden such as India. Furthermore, these findings provide a foundation for future studies exploring targeted therapeutic strategies and personalized management approaches in GB-OSCC.

## 5 Conclusion

This study demonstrates that integrated proteomic profiling combined with IHC validation can identify clinically relevant biomarkers associated with tumor aggressiveness in GB-OSCC. CA1, HBB, Torsin A, and *α*-Tubulin showed progressive overexpression from dysplasia to invasive carcinoma and were significantly associated with adverse histopathological features including tumor invasion, lymphovascular invasion, ITF characteristics, and tumor budding. To the best of the author’s knowledge, this is among the first studies to report the potential role of Torsin A, HBB, and *α*-Tubulin as candidate biomarkers in GB-OSCC. These findings support the potential utility of an immunohistochemistry-based biomarker panel for identifying oral potentially malignant disorders at risk of progression and for prognostic stratification of invasive OSCC. Further large-scale multicentric validation studies are required before clinical implementation.

## Data Availability

The original contributions presented in the study are included in the article/Supplementary material, further inquiries can be directed to the corresponding author/s.
