# Supplementary material for: Proteomic identification and histopathological validation of candidate biomarkers CA1, HBB, Torsin A, and α-Tubulin in gingivo-buccal oral squamous cell carcinoma
Source: Front Med (Lausanne). 2026 Aug 31;13:1843924. doi: 10.3389/fmed.2026.1843924 (PMC13572669; doi:10.3389/fmed.2026.1843924)
Supplement: Supplementary file 1 [file Table_1.docx]

**SUPPLEMENTARY**

**Table S1.** Demographic Characteristics of Patients Included in 2D Gel Electrophoresis Analysis

| **Group I** | | | | | | | | | | |
| --- | --- | --- | --- | --- | --- | --- | --- | --- | --- | --- |
| **Late-stage failure** | **Age** | **Gender** | **Tobacco chewing** | **No of yrs of Tobacco chewing** | **Smoking** | **Alcohol** | **TNM** | **HP Grade** | **Primary Treatment** | **Recurrence** |
| 1 | 32 | M | YES | 6 | NO | NO | T4aN2bM0 | PDSCC | CCR | LOCOREGIONAL |
| 2 | 55 | M | YES | 20 | YES | YES | T4aN1M0 | MDSCC | CCR | LOCOREGIONAL |
| 3 | 44 | F | YES | 12 | NO | NO | T4aN1M0 | MDSCC | Surgery | LOCAL |
| 4 | 56 | M | YES | 10 | NO | NIL | T4aN2bM0 | WDSCC | Surgery | LOCOREGIONAL |
| 5 | 57 | M | YES | 30 | NO | NO | T4aN2bM0 | MDSCC | Surgery | LOCAL |
| 6 | 55 | M | YES | 25 | YES | YES | T4aN2bM0 | MDSCC | Surgery | LOCAL |
| 7 | 55 | F | YES | 25 | YES | NO | T4aN1M0 | MDSCC | Surgery | LOCAL |
| 8 | 39 | M | YES | 15 | NO | NO | T4aN2BM0 | MDSCC | Surgery | LOCOREGIONAL |
| 9 | 59 | M | YES | 35 | NO | NO | T4aN1M0 | WDSCC | Surgery | LOCOREGIONAL |
| 10 | 42 | M | YES | 20 | NO | NO | T4aN1M0 | MDSCC | Surgery | LOCOREGIONAL |
| **Group II** | | | | | | | | | | |
| **Late-stage NED** | **Age** | **Gender** | **Tobacco chewing** | **No of yrs of Tobacco chewing** | **Smoking** | **Alcohol** | **TNM** | **HP Grade** | **Primary Treatment** | **Recurrence** |
| 1 | 59 | M | YES | 30 | NO | NO | T4aN1M0 | MDSCC | Surgery | NED |
| 2 | 26 | M | YES | 5 | NO | NO | T4aN0M0 | MDSCC | Surgery | NED |
| 3 | 71 | M | YES | 15 | NO | NO | T4aN0M0 | MDSCC | Surgery | NED |
| 4 | 47 | M | YES | 25 | NO | YES | T4aN1M0 | MDSCC | Surgery | NED |
| 5 | 45 | M | YES | 20 | YES | YES | T3N1M0 | MDSCC | Surgery | NED |
| 6 | 38 | M | YES | 10 | YES | NO | T4AN1M0 | MDSCC | Surgery | NED |
| 7 | 55 | F | YES | 20 | NO | NO | T4aN2BM0 | PDSCC | Surgery | NED |
| 8 | 48 | M | YES | 3 | NO | YES | T2N1M0 | MDSCC | Surgery | NED |
| 9 | 71 | M | YES | 15 | YES | NO | T4aN1M0 | WDSCC | Surgery | NED |
| 10 | 52 | F | YES | 25 | NO | NO | T4aN1M0 | WDSCC | Surgery | NED |
| **Group III** | | | | | | | | | | |
| **Early-stage NED** | **Age** | **Gender** | **Tobacco chewing** | **No of yrs of Tobacco chewing** | **Smoking** | **Alcohol** | **TNM** | **HP Grade** | **Primary Treatment** | **Recurrence** |
| 1 | 58 | M | YES | 22 | NO | YES | T1N0M0 | WDSCC | Surgery | NED |
| 2 | 59 | M | YES | 30 | NO | YES | T2N0M0 | PDSCC | Surgery | NED |
| 3 | 68 | M | YES | 5 | YES | NO | T2N0M0 | WDSCC | Surgery | NED |
| 4 | 59 | M | YES | 25 | YES | YES | T2NOMO | MDSCC | Surgery | NED |
| 5 | 64 | M | YES | 7 | NO | NO | T2N0M0 | WDSCC | Surgery | NED |
| 6 | 47 | F | YES | 14 | NO | NO | T2N0M0 | MDSCC | Surgery | NED |
| 7 | 36 | M | YES | 10 | YES | NO | T1N0M0 | MDSCC | Surgery | NED |
| 8 | 61 | M | YES | 35 | NO | YES | T2N0M0 | MDSCC | Surgery | NED |
| 9 | 52 | M | YES | 22 | YES | NO | T2N0M0 | MDSCC | Surgery | NED |
| 10 | 55 | F | YES | 20 | NO | NO | T1N0M0 | MDSCC | Surgery | NED |
| **Group IV** | | | | | | | | | | |
| **Early-stage failure** | **Age** | **Gender** | **Tobacco chewing** | **No of yrs of Tobacco chewing** | **Smoking** | **Alcohol** | **TNM** | **HP Grade** | **Primary Treatment** | **Recurrence** |
| 1 | 80 | F | YES | 50 | NO | NO | T2N0M0 | PDSCC | CCR | LOCOREGIONAL |
| 2 | 46 | F | YES | 10 | NO | NO | T2N0M0 | MDSCC | CCR | LOCOREGIONAL |
| 3 | 48 | M | YES | 10 | YES | 23 | T2N0M0 | WDSCC | Surgery | LOCAL |
| 4 | 47 | M | YES | 10 | YES | NO | T2N0M0 | MDSCC | Surgery | LOCAL |
| 5 | 44 | M | YES | 10 | YES | YES | T2N0M0 | MDSCC | Surgery | LOCAL |
| 6 | 71 | M | YES | 45 | YES | YES | T2N0M0 | MDSCC | Surgery | LOCAL |
| 7 | 39 | M | YES | 5 | YES | NO | T2N0M0 | WDSCC | Surgery | LOCOREGIONAL |
| 8 | 42 | M | YES | 15 | NO | NO | T1N0M0 | MDSCC | Surgery | LOCOREGIONAL |
| 9 | 56 | M | YES | 25 | YES | NO | T2N0M0 | MDSCC | Surgery | LOCAL |
| 10 | 64 | M | YES | 30 | NO | YES | T2N0M0 | MDSCC | Surgery | LOCAL |

**Table S2.** List of 37 differentially expressed upregulated proteins identified using MALDI-TOF/MS and their Mascot Scores, Sequence Coverage, Molecular Weights, and Isoelectric Points (pI).

| **S no** | **Match ID** | **Accession No** | **Protein Name** | Mascot Score | Coverage | Mol wt | Isoelectric Point  (pI) |
| --- | --- | --- | --- | --- | --- | --- | --- |
| 1 | 5 | HBB_HUMAN | Haemoglobin subunit beta OS=Homo sapiens OX=9606 GN=HBB PE=1 SV=2 | 104 | 70 | 16.102 | 6.75 |
| 2 | 6 | Q5NV90_HUMAN | V2-17 protein (Fragment) OS=Homo sapiens GN=IGLV3-25 PE=1 SV=1 - [Q5NV90_HUMAN] | 14 | 31 | 10.398 | 4.59 |
| 3 | 8 | ROP1A_HUMAN | Ropporin-1A OS=Homo sapiens OX=9606 GN=ROPN1 PE=1 SV=2 | 24 | 25 | 24.162 | 5.52 |
| 4 | 9 | RAB37_HUMAN | Ras-related protein Rab-37 OS=Homo sapiens OX=9606 GN=RAB37 PE=1 SV=3 | 57 | 49 | 25.028 | 5.97 |
| 5 | 10 | HSPB1_HUMAN | Heat shock protein beta-1 OS=Homo sapiens OX=9606 GN=HSPB1 PE=1 SV=2 | 46 | 36 | 22.826 | 5.98 |
| 6 | 15 | CAH1_HUMAN | Carbonic anhydrase 1 OS=Homo sapiens GN=CA1 PE=1 SV=2 - [CAH1_HUMAN] | 254 | 64 | 28.85 | 7.12 |
| 7 | 16 | CAH1_HUMAN | Carbonic anhydrase 1 OS=Homo sapiens OX=9606 GN=CA1 PE=1 SV=2 | 57 | 46 | 28.909 | 6.59 |
| 8 | 17 | CAH3_HUMAN | Carbonic anhydrase 3 OS=Homo sapiens GN=CA3 PE=1 SV=3 - [CAH3_HUMAN] | 43 | 55 | 29.53 | 7.34 |
| 9 | 18 | CAH2_HUMAN | Carbonic anhydrase 2 OS=Homo sapiens GN=CA2 PE=1 SV=2 - [CAH2_HUMAN] | 62 | 52 | 29.22 | 7.4 |
| 10 | 20 | B4DUJ3_HUMAN | cDNA FLJ52895, highly similar to Carbonic anhydrase 3 (EC 4.2.1.1) OS=Homo sapiens PE=2 SV=1 - [B4DUJ3_HUMAN] | 11 | 29 | 26.79 | 8.3 |
| 11 | 25 | G3P_HUMAN | Glyceraldehyde-3-phosphate dehydrogenase OS=Homo sapiens OX=9606 GN=GAPDH PE=1 SV=3 | 25 | 19 | 36.201 | 8.57 |
| 12 | 27 | E7EUT5_HUMAN | Glyceraldehyde-3-phosphate dehydrogenase OS=Homo sapiens GN=GAPDH PE=2 SV=1 - [E7EUT5_HUMAN] | 41 | 42 | 29 | 7 |
| 13 | 28 | TOR1A_HUMAN | Torsin-1A OS=Homo sapiens OX=9606 GN=TOR1A PE=1 SV=1 | 24 | 22 | 38.127 | 6.51 |
| 14 | 29 | BGH3_HUMAN | Transforming growth factor-beta-induced protein ig-h3 OS=Homo sapiens GN=TGFBI PE=2 SV=1 - [BGH3_HUMAN] | 140 | 38 | 74.64 | 7.25 |
| 15 | 33 | Q86VG2_HUMAN | Splicing factor proline/glutamine-rich (Polypyrimidine tract binding protein associated) OS=Homo sapiens GN=SFPQ PE=2 SV=1 - [Q86VG2_HUMAN] | 39 | 27 | 76.14 | 9.48 |
| 16 | 34 | K1C14_HUMAN | Keratin, type I cytoskeletal 14 OS=Homo sapiens GN=KRT14 PE=1 SV=4 - [K1C14_HUMAN] | 141 | 66 | 51.52 | 5.16 |
| 17 | 35 | H6VRG0_HUMAN | Keratin 1 OS=Homo sapiens GN=KRT1 PE=3 SV=1 - [H6VRG0_HUMAN] | 122 | 66 | 65.92 | 7.79 |
| 18 | 38 | K2C6A_HUMAN | Keratin, type II cytoskeletal 6A OS=Homo sapiens OX=9606 GN=KRT6A PE=1 SV=3 | 102 | 37 | 60.293 | 8.09 |
| 19 | 40 | K1C16_HUMAN | Keratin, type I cytoskeletal 16 OS=Homo sapiens GN=KRT16 PE=1 SV=4 - [K1C16_HUMAN] | 196 | 64 | 51.23 | 5.04 |
| 20 | 41 | E7EUT5_HUMAN | Glyceraldehyde-3-phosphate dehydrogenase OS=Homo sapiens GN=GAPDH PE=2 SV=1 - [E7EUT5_HUMAN] | 41 | 63 | 29 | 8 |
| 21 | 44 | ZN525_HUMAN | Zinc finger protein 525 OS=Homo sapiens OX=9606 GN=ZNF525 PE=2 SV=2 | 41 | 45 | 24.138 | 8.98 |
| 22 | 45 | A8K0L8_HUMAN | cDNA FLJ76998, highly similar to Homo sapiens zinc finger protein 28 homolog (mouse), mRNA OS=Homo sapiens OX=9606 PE=2 SV=1 | 73 | 22 | 38.9 | 9.77 |
| 23 | 48 | H0YA55_HUMAN | Serum albumin (Fragment) OS=Homo sapiens GN=ALB PE=4 SV=1 - [H0YA55_HUMAN] | 24 | 16 | 51.535 | 6.94 |
| 24 | 49 | ALBU_HUMAN | Serum albumin OS=Homo sapiens OX=9606 GN=ALB PE=1 SV=2 | 82 | 29 | 71.317 | 5.92 |
| 25 | 52 | TBA4A_HUMAN | Tubulin alpha-4A chain OS=Homo sapiens GN=TUBA4A PE=2 SV=1 - [A8MUB1_HUMAN] | 13 | 12 | 48.29 | 5 |
| 26 | 54 | K2C5_HUMAN | Keratin, type II cytoskeletal 5 OS=Homo sapiens OX=9606 GN=KRT5 PE=1 SV=3 | 124 | 35 | 62.568 | 7.59 |
| 27 | 55 | PRDX2_HUMAN | Peroxiredoxin-2 OS=Homo sapiens OX=9606 GN=PRDX2 PE=1 SV=5 | 48 | 34 | 22.049 | 5.66 |
| 28 | 58 | GDIR1_HUMAN | Rho GDP-dissociation inhibitor 1 OS=Homo sapiens GN=ARHGDIA PE=1 SV=3 - [GDIR1_HUMAN] | 69 | 45 | 23.19 | 5.1 |
| 29 | 60 | IF1AY_HUMAN | Eukaryotic translation initiation factor 1A, Y-chromosomal OS=Homo sapiens GN=EIF1AY PE=1 SV=4 - [IF1AY_HUMAN] | 55 | 37 | 16 | 4.2 |
| 30 | 62 | IF2A_HUMAN | Eukaryotic translation initiation factor 2 subunit 1 OS=Homo sapiens OX=9606 GN=EIF2S1 PE=1SV=3 | 24 | 28 | 36.374 | 5.02 |
| 31 | 63 | K22E_HUMAN | Keratin, type II cytoskeletal 2 epidermal OS=Homo sapiens GN=KRT2 PE=1 SV=2 - [K22E_HUMAN] | 242 | 86 | 65.4 | 8 |
| 32 | 65 | K2C6B_HUMAN | Keratin, type II cytoskeletal 6B OS=Homo sapiens OX=9606 GN=KRT6B PE=1 SV=5 | 59 | 24 | 60.315 | 8.09 |
| 33 | 66 | SMIM2_HUMAN | Small integral membrane protein 2 OS=Homo sapiens OX=9606 GN=SMIM2 PE=1 SV=1 | 32 | 41 | 9.628 | 5.19 |
| 34 | 68 | TPIS_HUMAN | Triosephosphate isomerase OS=Homo sapiens GN=TPI1 PE=1 SV=3 - [TPIS_HUMAN] | 100 | 70 | 30.8 | 5.92 |
| 35 | 78 | TBA1A_HUMAN | Tubulin alpha-1A chain OS=Homo sapiens GN=TUBA1A PE=3 SV=1 - [G3V1U9_HUMAN] | 88 | 45 | 46.3 | 5.08 |
| 36 | 80 | B4DW52_HUMAN | cDNA FLJ55253, highly similar to Actin, cytoplasmic 1 OS=Homo sapiens PE=2 SV=1 - [B4DW52_HUMAN] | 214 | 76 | 38.6 | 5.35 |
| 37 | 74 | B8ZWD1_HUMAN | Diazepam binding inhibitor, splice form 1A(2) OS=Homo sapiens GN=DBI PE=2 SV=1 - [B8ZWD1_HUMAN] | 129 | 82 | 11.1 | 8.32 |

s

**Table S3. Clinical and histopathological features Vs CA1 expression positive and negative groups among normal, dysplasia and GBC-SCC samples**

| **Criteria** | **Negative**  **(n=30)** | **Positive**  **(n=77)** | **Total**  **(n=107)** | **p value/ Significance** |
| --- | --- | --- | --- | --- |
| **Age Vs IHC for CA1** | | | | |
| Less than 35 | 12(60) | 8(40) | 20 | **p<0.002** |
| 36-60 | 12(21.1) | 45(78.9) | 57 |  |
| More then 60 | 6(20) | 24(80) | 30 |  |
| **Tobacco Chewing Vs IHC for CA1** | | | | |
| No | 13(52) | 12(48) | 25 | **p<0.010** |
| Commercial preparation | 14(20.6) | 54(79.4) | 68 |  |
| Quid | 3(21.4) | 11(78.6) | 14 |  |
| **Number of years of tobacco Chewing Vs IHC for CA1** | | | | |
| No tobacco chewing | 13(52) | 12(48) | 25 | **p**<**0.012** |
| 1-10yrs | 3(27.3) | 8(72.7) | 11 |  |
| 11-20yrs | 7(28) | 18(72) | 25 |  |
| >21yrs | 7(15.2) | 39(84.8) | 46 |  |
| **Tobacco Smoking Vs IHC for CA1** | | | | |
| No | 23(44.23) | 29(55.76) | 52 | **p**<**0.000** |
| Yes | 7(12.72) | 48(87.27) | 55 |  |
| **Number of years of Tobacco smoking Vs IHC for CA1** | | | | |
| No smoking | 23(44.23) | 29(55.76) | 52 | **p**<**0.002** |
| <10 yrs | 3(25) | 9(75) | 12 |  |
| 11-20 yrs | 3(15) | 17(85) | 20 |  |
| > 20 yrs | 1(4.34) | 22(95.65) | 23 |  |
| **Alcohol Vs IHC for CA1** | | | | |
| No | 25(40.32) | 37(59.67) | 62 | **p**<**0.001** |
| Yes | 5(11.11) | 40(88.88) | 45 |  |
| **All 3 habits Vs IHC for CA1** | | | | |
| No habits | 13(72.2) | 5(27.7) | 18 | p<**0.000** |
| 1. All 3 habits | 4(12.12) | 29(87.87) | 33 |  |
| 2.Chewing+Smoking | 3(23.07) | 10(76.92) | 13 |  |
| 3. Only chewing | 9(29.03) | 22(70.96) | 31 |  |
| 4.Chewing+Alcohol | 1(25) | 3(75) | 4 |  |
| 5.Smoking+Alcohol | 0 | 8(100) | 8 |  |
| **H/P Diagnosis Vs IHC for CA1** | | | | |
| Normal | 12(80) | 3(20) | 15 | **p**<**0.000** |
| Dysplasia | 12(38.70) | 19(61.29) | 31 |  |
| WDSCC | 4(11.11) | 32(88.88) | 36 |  |
| MDSCC | 2(8) | 23(92) | 25 |  |
| **H/P diagnosis 2 Vs IHC for CA1** | | | | |
| Normal | 12(80) | 3(20) | 15 | **p**<**0.000** |
| Mild dysplasia | 8(40) | 12(60) | 20 |  |
| Moderate/severe dysplasia | 4(36.36) | 7(63.63) | 11 |  |
| WDSCC | 4(11.11) | 32(88.88) | 36 |  |
| MDSCC | 2(8) | 23(92) | 25 |  |
| **Clinical Size Vs IHC for CA1** | | | | |
| Normal | 12(80) | 3(20) | 15 | **p**<**0.000** |
| Dysplasia | 12(38.70) | 19(61.29) | 31 |  |
| T1 | 1(33.33) | 2(66.66) | 3 |  |
| T2 | 2(7.69) | 24(92.30) | 26 |  |
| T3 | 3(14.28) | 18(85.71) | 21 |  |
| T4 | 0 | 11(100) | 11 |  |
| **Inflammatory cell infiltrate in Connective Tissue(CT) Vs IHC for CA1** | | | | |
| No inflammation | 5(83.3) | 1(16.6) | 6 | **p<0.009** |
| Mild | 7(22.5) | 24(77.41) | 31 |  |
| Moderate | 15(30.6) | 34(69.38) | 49 |  |
| Intense | 3(14.28) | 18(85.71) | 21 |  |
| **Invasion of muscle Vs IHC FOR CA1** | | | | |
| Normal/Dysplasia | 24(52.17) | 22(47.82) | 46 | **p**<**0.000** |
| no | 3(14.28) | 18(85.71) | 21 |  |
| yes | 3(7.5) | 37(92.5) | 40 |  |
| **Degree of Keratinization at ITF Vs IHC for CA1** | | | | |
| Normal/dysplasia | 24(52.17) | 22(47.82) | 46 | **p**<**0.000** |
| Degree of keratinization -1 | 3(42.85) | 4(57.1) | 7 |  |
| Degree of keratinization -2 | 1(5.55) | 17(94.44) | 18 |  |
| Degree of keratinization -3 | 1(4.347) | 22(95.65) | 23 |  |
| Degree of keratinization -4 | 1(7.69) | 12(92.30) | 13 |  |
| **Pattern of Invasion at ITF Vs IHC for CA1** | | | | |
| Normal/dysplasia | 24(52.17) | 22(47.82) | 46 | **p**<**0.000** |
| Invasion pattern 1 | 1(20) | 4(80) | 5 |  |
| Invasion pattern 2 | 3(13.63) | 19(86.36) | 22 |  |
| Invasion pattern 3 | 0 | 16(100) | 16 |  |
| Invasion pattern 4 | 2(11.11) | 16(88.88) | 18 |  |
| **Bryne’s grade at ITF Vs IHC for CA1** | | | | |
| Normal/Dysplasia | 24(52.17) | 22(47.82) | 46 | **p**<**0.000** |
| Low (4-8) | 4(25) | 12(75) | 16 |  |
| High (>8) | 2(4.44) | 43(95.55) | 45 |  |
| **Depth of invasion Vs IHC for CA1** | | | | |
| 0 | 24(52.17) | 22(47.82) | 46 | **p**<**0.000** |
| 1= >2000μm | 4(17.39) | 19(82.60) | 23 |  |
| 2 =2000-4000μm | 2(7.14) | 26(92.85) | 28 |  |
| 3=< 4000μm | 0 | 10(100) | 10 |  |
| **Lymphovascular Invasion Vs IHC for CA1** | | | | |
| Normal/dysplasia | 24(52.17) | 22(47.82) | 46 | **p**<**0.000** |
| No | 5(12.5) | 35(87.5) | 40 |  |
| Yes | 1(4.76) | 20(95.23) | 21 |  |
| **Tumor Budding Vs IHC for CA1** | | | | |
| Normal/dysplasia | 24(52.17) | 22(47.82) | 46 | **p**<**0.000** |
| Yes | 1(7.69) | 12(92.30) | 13 |  |
| No | 5(10.41) | 43(89.58) | 48 |  |

**Table S4. Clinical and histopathological features Vs HBB expression among normal, dysplasia and GBC-SCC samples.**

| **Criteria** | **Negative**  **n=40 (37.4%)** | **Mild n=21(19.6%)** | **Moderate**  **n= 13 (12.1%)** | **Intense n=33 (30.8%)** | **Total n= 107** | **p value/ Significance** |
| --- | --- | --- | --- | --- | --- | --- |
| **H/P Diagnosis Vs IHC FOR HBB** | | | | | | |
| Normal | 9(81.81) | 2(18.18) | 0 | 0 | 11 | **p<0.000** |
| Dysplasia | 17(62.96) | 6(22.22) | 4(14.81) | 0 | 27 |  |
| WDSCC | 8(21.05) | 8(21.05) | 5(13.15) | 17(44.73) | 38 |  |
| MDSCC | 6(19.35) | 5(16.1) | 4(12.903) | 16(51.61) | 31 |  |
| **H/P diagnosis 2 Vs IHC FOR HBB** | | | | | | |
| Normal | 9(81.81) | 2(18.18) | 0 | 0 | 11 | **p<0.000** |
| Hyperplasia+ Mild dysplasia | 15(75) | 4(20) | 1(5) | 0 | 20 |  |
| Moderate/severe dysplasia | 2(28.57) | 2(28.57) | 3(42.85) | 0 | 7 |  |
| WDSCC | 8(21.05) | 8(21.05) | 5(13.15) | 17(44.73) | 38 |  |
| MDSCC | 6(19.35) | 5(16.1) | 4(12.903) | 16(51.61) | 31 |  |
| **Clinical size VS IHC FOR HBB** | | | | | | |
| Normal | 9(81.81) | 2(18.18) | 0 | 0 | 11 | **p<0.000** |
| Dysplasia | 17(60.71) | 6(21.42) | 4(14.28) | 0 | 27 |  |
| T1 | 6(42.8571) | 5(35.71) | 2(7.14) | 2(14.28) | 15 |  |
| T2 | 4(16.66) | 5(20.833) | 5(20.833) | 10(41.66) | 24 |  |
| T3 | 3(25) | 0 | 1(8.33) | 8(66.66) | 12 |  |
| T4 | 1(5.55) | 3(16.66) | 2(11.11) | 12(66.66) | 18 |  |
| **Invasion of muscle Vs IHC FOR HBB** | | | | | | |
| Normal/Dysplasia | 26(68.42) | 8(21.05) | 4(10.52) | 0 | 38 | **p<0.000** |
| yes | 11(20.75) | 10(18.86) | 5(9.433) | 27(50.94) | 53 |  |
| no | 3(18.75) | 3(18.75) | 4(25) | 6(37.5) | 16 |  |
| **Degree Keratinization at ITF Vs IHC FOR HBB** | | | | | | |
| Normal/dysplasia | 26(68.42) | 8(21.05) | 4(10.52) | 0 | 38 | **p<0.000** |
| Degree Keratinization -1 | 1(25) | 0 | 1(25) | 2(50) | 4 |  |
| Degree Keratinization -2 | 4(25) | 5(31.25) | 2(12.5) | 5(31.25) | 16 |  |
| Degree Keratinization -3 | 4(13.33) | 5(16.66) | 4(13.33) | 17(56.66) | 30 |  |
| Degree Keratinization -4 | 5(26.31) | 3(15.78) | 2(10.52) | 9(47.36) | 19 |  |
| **Pattern of Invasion at ITF Vs IHC FOR HBB** | | | | | | |
| Normal/dysplasia | 26(68.42) | 8(21.05) | 4(10.52) | 0 | 38 | **p<0.000** |
| Pattern of invasion-1 | 1(100) | 0 | 0 | 0 | 1 |  |
| Pattern of invasion-2 | 6(37.5) | 3(18.75) | 1(6.25) | 6(37.5) | 16 |  |
| Pattern of invasion-3 | 1(4.54) | 3(13.63) | 5(22.72) | 13(59.09) | 22 |  |
| Pattern of invasion-4 | 6(20) | 7(23.33) | 3(10) | 14(46.66) | 30 |  |
| **Bryne’s grade at ITF vs IHC FOR HBB** | | | | | | |
| Normal/Dysplasia | 26(68.42) | 8(21.05) | 4(10.52) | 0 | 38 | **p<0.000** |
| Low Grade (4-8) | 5(41.66) | 2(16.66) | 2(16.66) | 3(25) | 12 |  |
| High Grade (>8) | 9(15.78) | 11(19.29) | 7(12.28) | 30(52.63) | 57 |  |
| **Depth of Invasion Vs IHC FOR HBB** | | | | | | |
| Normal/Dysplasia | 26(68.42) | 8(21.05) | 4(10.52) | 0 | 38 | **p<0.000** |
| 1= >2000μm | 7(36.84) | 3(15.78) | 2(10.52) | 7(36.84) | 19 |  |
| 2 =2000-4000μm | 5(16.66) | 5(16.66) | 4(13.33) | 16(53.33) | 30 |  |
| 3=< 4000μm | 2(10) | 5(25) | 3(15) | 10(50) | 20 |  |
| **Lymph vascular Invasion Vs IHC FOR HBB** | | | | | | |
| Normal/ dysplasia | 26(68.42) | 8(21.05) | 4(10.52) | 0 | 38 | **p<0.000** |
| Yes | 4(19.04) | 4(19.04) | 2(9.52) | 11(52.38) | 21 |  |
| No | 10(20.8) | 9(18.8) | 7(14.6) | 22(45.8) | 48 |  |
| **Tumor Budding Vs IHC FOR HBB** | | | | | | |
| Normal/dysplasia | 26(68.42) | 8(21.05) | 4(10.52) | 0 | 38 | **p<0.000** |
| Yes | 6(21.42) | 6(21.42) | 1(3.57) | 15(53.57) | 28 |  |
| No | 8(19.51) | 7(17.07) | 8(19.51) | 18(43.90) | 41 |  |

**Table S5. Clinical and histopathological features Vs Torsin-A** **expression among normal, dysplasia and GBC-SCC samples.**

| **Criteria** | **Negative**  **n=11** | **Mild n=1** | **Moderate**  **n= 5** | **Intense n= 89** | **Total n= 106** | **p value/ Significance** |
| --- | --- | --- | --- | --- | --- | --- |
| **H/P diagnosis Vs IHC for Torsin-A** | | | | | | |
| Normal | 3(30) | 1(10) | 0 | 6(60) | 10 | **p<0.000** |
| Dysplasia | 8(25.80) | 0 | 1(3.22) | 22(70.96) | 31 |  |
| WDSCC | 0 | 0 | 2(6.89) | 27(93.10) | 29 |  |
| MDSCC | 0 | 0 | 2(5.55) | 34(94.44) | 36 |  |
| **H/P diagnosis 2 Vs IHC for Torsin-A** | | | | | | |
| Normal | 3(30) | 1(10) | 0 | 6(60) | 10 | **p<0.003** |
| Mild dysplasia | 5(26.31) | 0 | 1(5.26) | 13(68.42) | 19 |  |
| Moderate/severe dysplasia | 3(25) | 0 | 0 | 9(75) | 12 |  |
| WDSCC | 0 | 0 | 2(6.89) | 27(93.10) | 29 |  |
| MDSCC | 0 | 0 | 2(5.55) | 34(94.44) | 36 |  |
| **Clinical Size Vs H/P diagnosis Vs IHC for Torsin-A** | | | | | | |
| Normal | 3(30) | 1(10) | 0 | 6(60) | 10 | **p<0.012** |
| Dysplasia | 7(22.58) | 0 | 1(3.22) | 23(74.19) | 31 |  |
| T1 | 0 | 0 | 0 | 4(100) | 4 |  |
| T2 | 0 | 0 | 2(7.69) | 24(92.3) | 26 |  |
| T3 | 1(5.26) | 0 | 0 | 18(94.73) | 19 |  |
| T4 | 0 | 0 | 2(12.5) | 14(87.5) | 16 |  |
| **Invasion of muscle Vs IHC for Torsin-A** | | | | | | |
| Normal/dysplasia | 11(26.829) | 1(2.43) | 1(2.43) | 28(68.29) | 41 | **p<0.000** |
| no | 0 | 0 | 0 | 20(100) | 20 |  |
| yes | 0 | 0 | 4(8.88) | 41(91.11) | 45 |  |
| **Degree of keratinization at ITF Vs IHC for Torsin-A** | | | | | | |
| Normal/dysplasia | 11(26.829) | 1(2.43) | 1(2.43) | 28(68.29) | 41 | **p<0.000** |
| Degree of keratinization -1 | 0 | 0 | 2(33.33) | 4(66.66) | 6 |  |
| Degree of keratinization -2 | 0 | 0 | 0 | 19(100) | 19 |  |
| Degree of keratinization -3 | 0 | 0 | 0 | 24(100) | 24 |  |
| Degree of keratinization -4 | 0 | 0 | 2(12.5) | 14(87.5) | 16 |  |
| **Pattern of Invasion at ITF Vs IHC for Torsin-A** | | | | | | |
| Normal/dys | 11(26.829) | 1(2.43) | 1(2.43) | 28(68.29) | 41 | **p<0.001** |
| Pattern of Invasion**-**1 | 0 | 0 | 2(33.33) | 4(66.66) | 6 |  |
| Pattern of Invasion**-**2 | 0 | 0 | 0 | 21(100) | 21 |  |
| Pattern of Invasion**-**3 | 0 | 0 | 2(8) | 23(92) | 25 |  |
| Pattern of Invasion**-**4 | 0 | 0 | 0 | 13(100) | 13 |  |
| **Bryne’s grade at ITF vs IHC for Torsin-A** | | | | | | |
| Normal/Dysplasia | 11(26.829) | 1(2.43) | 1(2.43) | 28(68.29) | 41 | **p<0.000** |
| Low Grade(4-8) | 0 | 0 | 2(14.28) | 12(85.71) | 14 |  |
| High Grade (>8) | 0 | 0 | 2(3.92) | 49(96.07) | 51 |  |
| **Depth of Invasion Vs IHC for Torsin-A** | | | | | | |
| Normal/Dysplasia | 11(26.829) | 1(2.43) | 1(2.43) | 28(68.29) | 41 | **p<0.004** |
| 1= >2000 μm | 0 | 0 | 1(4.34) | 22(95.65) | 23 |  |
| 2 =2000-4000 μm | 0 | 0 | 3(10.71) | 25(89.28) | 28 |  |
| 3=< 4000 μm | 0 | 0 | 0 | 14(100) | 14 |  |
| **Lymphovascular invasion Vs IHC for Torsin-A** | | | | | | |
| Normal/dysplasia | 11(26.829) | 1(2.43) | 1(2.43) | 28(68.29) | 41 | **p<0.001** |
| Yes | 0 | 0 | 2(8) | 23(92) | 25 |  |
| No | 0 | 0 | 2(5) | 38(95) | 40 |  |
| **Tumor Budding Vs IHC for Torsin-A** | | | | | | |
| Normal/dys | 11(26.829) | 1(2.43) | 1(2.43) | 28(68.29) | 41 | **p<0.001** |
| Yes | 0 | 0 | 0 | 11(100) | 11 |  |
| No | 0 | 0 | 4(7.40) | 50(92.59) | 54 |  |

**Table S6. Clinical and histopathological features Vs α-Tubulin expression among normal, dysplasia and GBC-SCC samples.**

| **Criteria** | **Negative**  **n=11** | **Mild**  **n=4** | **Moderate**  **n= 5** | **Intense n=86** | **Total = 106** | **p value/ Significance** |
| --- | --- | --- | --- | --- | --- | --- |
| **H/P diagnosis Vs IHC for *α-*Tubulin** | | | | | | |
| Normal | 4(40) | 0 | 0 | 6(60) | 10 | **p<0.001** |
| dysplasia | 7(22.6) | 1(3.2) | 0 | 23(74.2) | 31 |  |
| WDSCC | 0 | 2(6.89) | 1(3.44) | 26(89.65) | 29 |  |
| MDSCC | 0 | 0 | 4(11.42) | 31(88.57) | 35 |  |
| **H/P diagnosis 2 Vs IHC for *α-*Tubulin** | | | | | | |
| Normal | 4(40) | 0 | 0 | 6(60) | 10 | **p<0.002** |
| Mild dysplasia | 4(21.05) | 0 | 0 | 15(78.94) | 19 |  |
| Moderate/severe dysplasia | 2(16.7) | 1(8.3) | 0 | 9(75) | 12 |  |
| WDSCC | 0 | 2(6.89) | 1(3.44) | 26(89.65) | 29 |  |
| MDSCC | 0 | 0 | 4(11.42) | 31(88.57) | 35 |  |
| **Clinical size Vs IHC for *α-*Tubulin** | | | | | | |
| Dysplasia | 7(22.58) | 0 | 0 | 24(77.41) | 31 | **p<0.006** |
| Normal | 4(40) | 0 | 0 | 6(60) | 10 |  |
| T1 | 0 | 0 | 0 | 4(100) | 4 |  |
| T2 | 0 | 3(10.71) | 3(10.71) | 22(78.57) | 28 |  |
| T3 | 0 | 1(5.26) | 1(5.26) | 17(89.47) | 19 |  |
| T4 | 0 | 0 | 1(6.25) | 15(93.75) | 16 |  |
| **Invasion of muscle Vs IHC for *α-*Tubulin** | | | | | | |
| Normal/Dysplasia | 11(26.89) | 1(2.43) | 0 | 29(70.73) | 41 | **p<0.000** |
| No | 0 | 2(10) | 1(5) | 17(85) | 20 |  |
| Yes | 0 | 1(2.22) | 4(8.88) | 40(88.88) | 45 |  |
| **Degree of keratinization at ITF Vs IHC for *α-*Tubulin** | | | | | | |
| Normal/ dysplasia | 11(26.89) | 1(2.43) | 0 | 29(70.73) | 41 | **p<0.001** |
| Degree of keratinization -1 | 0 | 1(16.66) | 0 | 5(83.33) | 6 |  |
| Degree of keratinization -2 | 0 | 1(5.26) | 0 | 18(94.7) | 19 |  |
| Degree of keratinization -3 | 0 | 1(4.16) | 2(8.33) | 21(87.5) | 24 |  |
| Degree of keratinization -4 | 0 | 0 | 3(18.75) | 13(81.25) | 16 |  |
| **Pattern of Invasion at ITF Vs IHC for *α-*Tubulin** | | | | | | |
| Normal/ dysplasia | 11(26.89) | 1(2.43) | 0 | 29(70.73) | 41 | **p<0.007** |
| Pattern of Invasion-1 | 0 | 1(16.66) | 0 | 5(83.33) | 6 |  |
| Pattern of Invasion-2 | 0 | 1(4.76) | 1(4.76) | 19(90.47) | 21 |  |
| Pattern of Invasion-3 | 0 | 1(4) | 3(12) | 21(84) | 25 |  |
| Pattern of Invasion-4 | 0 | 0 | 1(7.69) | 12(92.30) | 13 |  |
| **Bryne’s grade at ITF vs IHC for *α-*Tubulin** | | | | | | |
| Normal/Dysplasia | 11(26.89) | 1(2.43) | 0 | 29(70.731) | 41 | **p<0.006** |
| Low Grade(4-8) | 0 | 1(7.14) | 0 | 13(92.857) | 14 |  |
| High Grade (>8) | 0 | 2(3.92) | 5(9.80) | 44(86.27) | 51 |  |
| **Depth of Invasion Vs IHC for *α-*Tubulin** | | | | | | |
| Normal/dysplasia | 11(26.82) | 1(2.43) | 0 | 29(70.73) | 41 | **p<0.006** |
| 1= >2000 μm | 0 | 1(4.34) | 2(8.69) | 20(86.95) | 23 |  |
| 2 =2000-4000 μm | 0 | 2(7.14) | 2(7.14) | 24(85.71) | 28 |  |
| 3=< 4000 μm | 0 | 0 | 1(7.14) | 13(92.85) | 14 |  |
| **Lymphovascular invasion Vs IHC for *α-*Tubulin** | | | | | | |
| Normal/dysplasia | 11(26.82) | 1(2.43) | 0 | 29(70.73) | 41 | **p<0.000** |
| Yes | 0 | 0 | 3(12) | 22(88) | 25 |  |
| No | 0 | 3(7.5) | 2(5) | 35(87.5) | 40 |  |
| **Tumor Budding Vs IHC for *α-*Tubulin** | | | | | | |
| Normal/dysplasia | 11(26.82) | 1(2.43) | 0 | 29(70.73) | 41 | **p<0.001** |
| Yes | 0 | 1(9.09) | 1(9.09) | 9(81.81) | 11 |  |
| No | 0 | 2(3.703) | 4(7.40) | 48(88.88) | 54 |  |

|  | binding (GO:0005488) |
| --- | --- |
|  | catalytic activity (GO:0003824) |
|  | molecular function regulator (GO:0098772) |
|  | structural molecule activity (GO:0005198) |
|  | translation regulator activity (GO:0045182) |
|  | transporter activity (GO:0005215) |

**A)**

|  | biological adhesion (GO:0022610) |
| --- | --- |
|  | biological regulation (GO:0065007) |
|  | cellular process (GO:0009987) |
|  | immune system process (GO:0002376) |
|  | localization (GO:0051179) |
|  | metabolic process (GO:0008152) |
|  | response to stimulus (GO:0050896) |
|  | signaling (GO:0023052) |

**B)**

|  | cellular anatomical entity (GO:0110165) |
| --- | --- |
|  | intracellular (GO:0005622) |
|  | protein-containing complex (GO:0032991) |

**C)**

**Figure S1.** Functional classification of 37 differentially regulated proteins and biological network analysis using PANTHER gene ontology database, were functionally categorized as based on: A) Molecular Function, B) Biological Process, C) Cellular Component

|  | cell adhesion molecule (PC00069) |
| --- | --- |
|  | chaperone (PC00072) |
|  | cytoskeletal protein (PC00085) |
|  | gene-specific transcriptional regulator (PC00264) |
|  | metabolite interconversion enzyme (PC00262) |
|  | nucleic acid metabolism protein (PC00171) |
|  | protein-binding activity modulator (PC00095) |
|  | transfer/carrier protein (PC00219) |
|  | translational protein (PC00263) |

**A)**

|  | Angiogenesis (P00005) |
| --- | --- |
|  | Apoptosis signaling pathway (P00006) |
|  | CCKR signaling map (P06959) |
|  | Glycolysis (P00024) |
|  | Gonadotropin-releasing hormone receptor pathway (P06664) |
|  | Huntington disease (P00029) |
|  | Parkinson disease (P00049) |
|  | VEGF signaling pathway (P00056) |
|  | p38 MAPK pathway (P05918) |

**B)**

**Figure S2.** A) Protein classification and B) pathway analysis using PANTHER database for 37 differentially upregulated proteins
